# The last builders of megaliths in the South of the Iberian peninsula? The Bronze Age necropolis of Trafalgar (Barbate, Cádiz, Spain)

**DOI:** 10.1371/journal.pone.0359220

**Published:** 2026-11-11

**Authors:** Eduardo Vijande-Vila, Serafín Becerra-Martín, Berta Morell-Rovira, Marta Díaz-Zorita Bonilla, Eduardo Molina-Piernas, Alejandro Muñoz-Muñoz, Adolfo Moreno-Márquez, Pablo González-Fernández, Salvador Domínguez-Bella, María Leticia Gómez-Sánchez, Diego Salvador Fernández-Sánchez, José Luis Ramírez-Amador, José María Corona-Borrego, María Carmona-Gil, Jesús Corrales Díaz, Juan Jesús Cantillo-Duarte

**Affiliations:** 1 Departamento de Historia, Geografía y Filosofía, Universidad de Cádiz, Cádiz, Spain; 2 Museo de Teba, Ayuntamiento de Teba, Málaga, Spain; 3 Seminario de Estudios e Investigaciones Prehistóricas (SERP), Universidad de Barcelona, Barcelona, Spain; 4 Departamento de Prehistoria y Arqueología, Universidad de Granada, Granada, Spain; 5 Institut für Ur- und Frühgeschichte und Archäologie des Mittelalters, University of Tübingen, Tübingen, Germany; 6 Departamento de Ciencias de la Tierra, Universidad de Cádiz, Cádiz, Spain; 7 Departamento de Geografía, Historia y Humanidades, Universidad de Almería, Almería, Spain; 8 Departamento de Prehistoria, Historia Antigua y Arqueología, Universidad Complutense de Madrid, Madrid, Spain; 9 Professional archaeologist, Andalucía, Spain; Vilnius University: Vilniaus Universitetas, LITHUANIA

## Abstract

The archaeological excavations undertaken at the Trafalgar tombolo during 2021–2022 led to the identification of a Bronze Age megalithic necropolis. This paper presents an interdisciplinary study, considering chronological, structural, bioarchaeological, and archaeometrical evidence of two megalithic tombs. The main aim of this work is to date both tombs through the use of Bayesian statistical models that confirms the use of the necropolis during the first quarter of the 2^nd^ millennium BC. Another goal is to define the period of use of these structures and the burial practices adopted by this community. Although the 2^nd^ millennium BC is characterized by the reuse of megalithic tombs from earlier periods, the significance of the Trafalgar necropolis also lais in the possibility to have been built *ex novo* during the first quarter of the 2^nd^ millennium BC. This would make it one of the latest megalithic necropolises of the Iberian peninsula. The use of high value objects such as gold, silver, and ivory, as grave goods and the necropolis’ location in an outstanding geographical landmark, Cape Trafalgar, suggests that the sea played a key role for these communities both economically (trade routes) and ideologically (as a funerary landscape). Finally, in addition to helping us with the architectural analysis of the two tombs, the use of new technologies (3D scanner and photogrammetry) allowed us to generate digital models for the transfer of knowledge purpose, since the site is challenging in terms of public value and recognition.

## 1. Introduction

Funerary practices in southern Iberia during the Bronze Age are characterised by great variability, with the emergence of new burial practices – individual cist burials– and the continued construction and/or reuse of megalithic structures inspired by the collective rituals that marked the Chalcolithic norm [1–4].

Traditionally, and too simplistically, it has been argued that, in the south-eastern Iberian peninsula, the emergence of El Argar [5] triggered a series of social and economic changes that led to a strongly stratified state structure, which in terms of burial customs involved the substitution of individual tombs for collective ones. A broader view of the funerary practices in place in Argaric territory has shown that collective megalithic burials persisted, perhaps as a strategy of resistance against the drive towards differentiation and individualization pushed by the Argaric society [3].

The reuse of megalithic monuments has been documented in the south-west of the peninsula, although the phenomenon is still poorly understood, because the existing examples are yet to be systematically reviewed and many lack radiocarbon dates. Although different necropolises with relative chronologies duringg the late 2^nd^ millennium BC have been excavated, few have solid series of radiocarbon dates [2,6–9]. In this sense, the Trafalgar necropolis stands as an exceptional example, presenting crucial data for the continuity of the megalithic phenomenon during the 2^nd^ millennium BC in the south of the Iberian peninsula.

The main goal of this study is to add to the existing knowledge on this final stage of Megalithism, with particular emphasis on: (1) establishing a safe chronology, to determine the persistence of megalithic funerary practices into the Bronze Age and the period of use of these tombs; (2) defining the architectural features that characterise the final stage of Megalithism; (3) gaining a better understanding of the role that the sea played for these societies (as liminal space and as trade route for high value goods, such as silver, gold, ivory, etc.); and (4) developing preservation and dissemination strategies for our megalithic heritage. With this purpose, we present a wide range of radiocarbon dates of human bone (short life samples), to which we have applied Bayesian statistical analysis. This is part of an interdisciplinary study of the architectural, anthropological, and archaeometric evidence, including the generation of digital models for a better understand of the continuity of megalithic funerary practices during the Bronze Age, and to preserve and value this specific heritage.

## 2. Archaeological background: The megalithic Necropolis of Trafalgar

This necropolis is located in Cape Trafalgar, known for the naval battle hosted in 1805. It is located in a hill from which a double tombolo (to the east and west) extends, connecting the hill to the mainland (Fig 1). Its unique orography, with a lagoon-like interior space, makes it obtain more of a rocky island, made of sandstone and shell conglomerates on which fossil and mobile dunes have formed [10–12]. Tombolos are unusual coastal morphology that happen when an island close to the coast connects with the continent with, at least, a sand strip. This space is located in the Atlantic shore of Cadiz, in the middle of the Strait of Gibraltar and just 44 km from the African continent.

**Fig 1:**
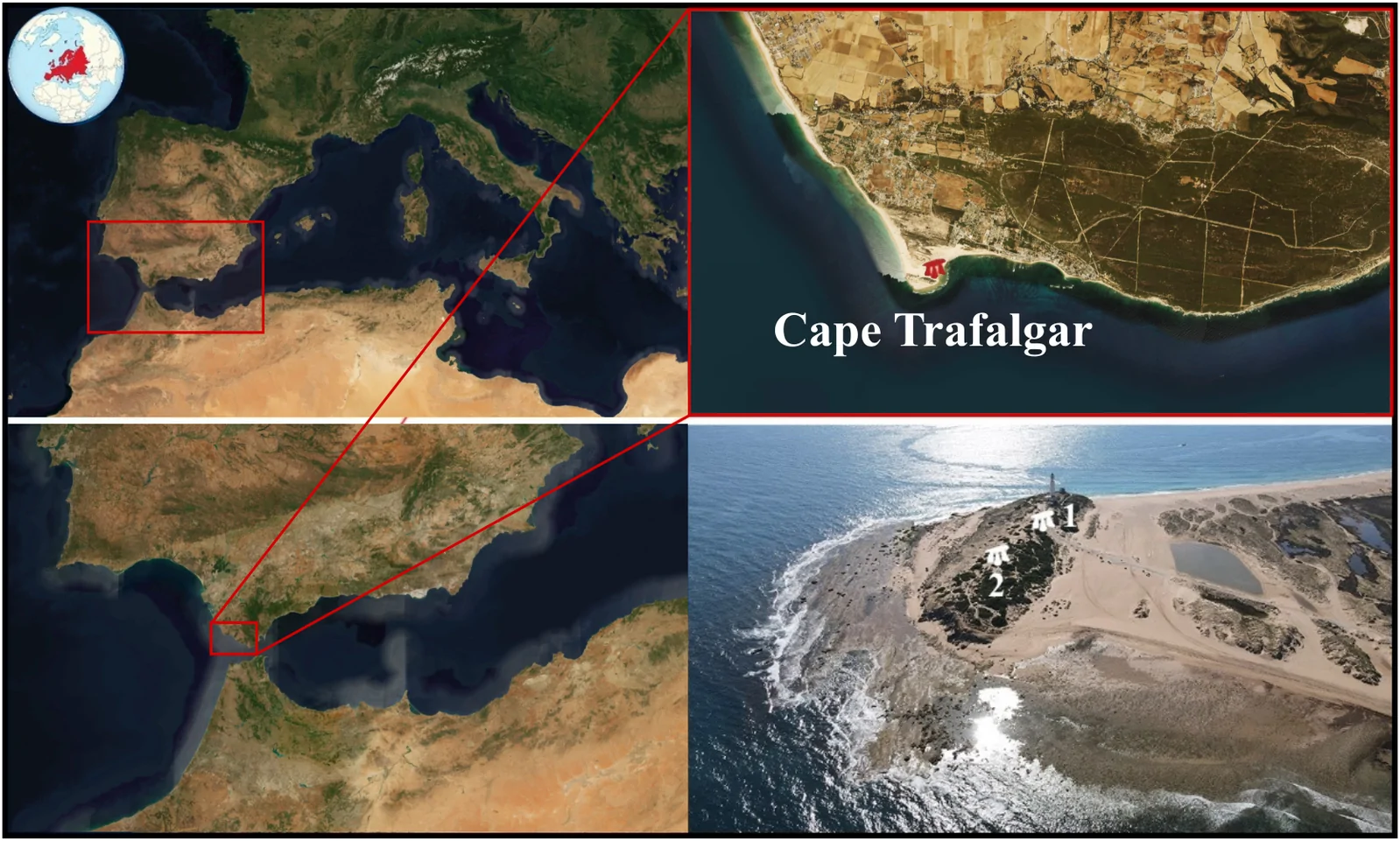
Geographical setting of the Necropolis of Trafalgar with indication of documented and excavated tombs. Satellite images were obtained using OpenStreetMap (https://www.openstreetmap.org/copyright) combined with orthophotos from the Spanish National Aerial Orthophotography Program (PNOA), provided as open data by the Spanish National Geographic Institute (IGN) under a CC BY 4.0 compatible license (https://centrodedescargas.cnig.es). The figure was designed using Adobe Photoshop version CC 2021 (22.3.1) (http://www.adobe.com).

Archaeological survey was carried out in Barbate in 2000, within the framework of the General Research Project *La ocupación prehistórica de la campiña litoral y banda atlántica de Cádiz*, allowed the documentation of ceramic and lithic finds dated to the early 2^nd^ millennium BC in the tombolo of Tragalfar, as well as stone tools dated to the Lower-Middle Palaeolithic period. In addition, the presence of “a cist tomb” virtually covered by a dune [13] was also detected. In May 2021, our team cleared this structure; although it originally seemed like a cist, it ended up being the initial section of the corridor of a megalithic tomb of greater complexity, which was designed as Tomb I. This construction presents a mixed typology (Rivero, 1988), with an orthostatic corridor that leads through a funerary chamber (or hypogeum) excavated in the geological substratum [14].

Later, in May 2022, we located and excavated a new funerary structure, roughly 180 m to the southeast of Tomb I (Fig 1). At the beginning of the excavation, only the top surface of the orthostates that formed the beginning of the corridor of Tomb II was visible. However, the removal of a dune, approximately 3 m high, revealed a funerary *tholos*-like structure, formed by a small access corridor that leads to a circular chamber, with a south-north orientation. Everything suggests that these two megalithic tombs are part of a larger necropolis, hidden by the dune on the tombolo [14]. It cannot be ruled out that future geophysical surveys can locate new tombs, outlining the boundaries of the necropolis, although we are aware of the difficulty that the low conductivity of dune sands poses for the use of this type of techniques in coastal environments [15].

## 3. Materials and methods

### 3.1 Architectural analysis

The architectural analysis of both megalithic tombs was undertaken with the support of digital documentation technologies, such as terrestrial laser scanner (TLS) and manual and aerial photogrammetry using drones, which made it possible to compile highly precise spatial data to a sub-centimetre resolution. The combined graphic resolution of laser scanner and photogrammetry allowed for the generation of a highly detailed planimetric survey, as well as high-resolution digital twins, which play a key role in the morphostructural interpretation of the features and the creation of educational visual resources.

Since both structures were again backfilled after excavation for preservation purposes, these digital models have allowed us to obtain plans, sections, digital elevations terrain models, etc*.* Furthermore, the use of software tools such as Blender aids in the generation of 3D models that can facilitate the dissemination and valorisation of buried archaeological sites on open-access platforms like Sketchfab (https://sketchfab.com/3d-models/tumba-ii-necropolis-de-trafalgar-12b6f593339c41089af7ba31f5808a87).

The technical equipment used consists of a 3D Leica RTC360 laser scanner, capable of acquiring 2 million data points per second and equipped with an advanced HDR image capture system. The device is also able to generate a 3D point cloud in under 2 minutes, and this can be previewed on a mobile device. The laser scanner was set up in between 20–23 positions per feature, guaranteeing full coverage with no critical overlaps. This strategy yielded highly precise results, with a recorded average error of 0.004 meters, ensuring an excellent level of detail for later analyses and preservation.

At the same time, a Nikon D7500 camera was used, equipped with a DX (APS-C) format CMOS sensor of 20.9 megapixels. This camera offers a maximum resolution of 5.568 x 3.712 pixels and allows a native ISO range of 100–51.

For the aerial photography, a DJI Mini 2 drone was used, equipped with a CMOS sensor of 1/ 2.3 inches and 12 effective megapixels. Its lens has an 83º field of view, equivalent to a focal length of 24 mm in full-frame format, with a fixed aperture of f/2.8. The drone can capture images in JPEG and RAW (DNG) formats, with a maximum resolution of 4,000 × 3,000 pixels. It is also able to record a 4K at 30 fps video, which provides detailed and high-quality aerial documentation.

For the processing and alignment of point clouds generated though laser scanner, Cyclone Register 360 (v. 2025.0.0) and Cyclone 3DR (v. 2021.0) software were used, both developed by Leica Geosystems. These tools allow for the efficient managing of vast volumes of data, as well as cleaning, aligning, and exporting georeferenced three-dimensional models.

The scanning of Tomb I yielded a disperse point cloud (18k points), a dense point cloud (20 million points), and a mesh (6 million polygons and approximately 2 million vertices). Texture size: 8192x8192 MP. Digital Elevation Model with a resolution of 3927x2841, 4.58 mm/pixel.

Tomb II yielded a disperse point cloud (345,398 points). In this instance, we used depth maps to produce a mesh (369,521,389 polygons and 185,027,886 vertices). Texture size: 8192x8192 MP.

The generation of photogrammetric models from terrestrial and aerial images was done with Agisoft Metashape Professional (v. 2.1.2) software, which allows for the dense reconstruction of surfaces and high-quality texturing, integrating both visual data and control points for accurate georeferencing.

### 3.2. Bioarchaeology

The inventoring of the commingled human remains has been done based on the classification of bone elements, teeth, and fragments following Brothwell [16], Schaefer et al. [17] and White & Folkens [18]. The fragmentation of the remains was recorded following the anatomical zone system developed by Knüsel & Outram [19]; this was necessary to estimate the minimum number of individuals (MNI) [20–22]. Aditionally, refitting exercises were carried out to ensure the accuracy of this method [23]. The estimation of the sexual characters of the bone elements was established using Ubelaker [24] and Buikstra & Ubelaker [25]. The age-at-death of the individuals has been established according to cranial suture closure [25], dental eruption phases [26], dental wear [27] or epiphysis fusion [28]. The skeletal pathologies were also identified [29].

### 3.3. Radiocarbon dates and Bayesian analysis

A total of 16 new radiocarbon dates were performed from human bone samples corresponding to 16 different individuals (Table 1). The sampling strategy for radiocarbon dating in Tomb I was focused following the MNI: a minimum number of 13 individuals was established based on mandibles, right scapulae, and upper right canines (FDI 13). Therefore, 10 samples were taken from bone mandibles or their associated dentition from Tomb I (representing 76.92% of the minimum number of individuals). At Tomb II, the sampling strategy focused on osseous sets (bone accumulation) observed during the excavation. The subsequent study of bone remains reinforces our hypothesis that each of these depositions (sets) probably belong to a single individual, since there is no repetition of osseous elements in the remains corresponding to each set. Each of the sampled bone acumulations contained a left zygomatic process; this element gives us the highest MNE for Tomb II, stablishing an MNI of 7 for this tomb. Samples were taken from the dentition of the skulls associated with each set, with a total of six teeth (Table 1).

**Table 1:** Radiocarbon dates of the Tomb I and Tomb II of Trafalgar. Calibrations were performed in OxCal v4.4 using the IntCal20 calibration curve.

Lab Code	Radiocarbon age (¹⁴C yr BP)	SD	C/N	Tomb	Individual	Sample	cal BC, 95.4% probability	Location
SUERC110533	3503	30	3.4	I	NA	Mandible frag.	1920−1742	Chamber
SUERC110532	3483	30	3.3	I	NA	Mandible frag	1890−1695	Chamber
SUERC110539	3468	23	3.5	I	NA	Mandible frag	1880−1695	Chamber
SUERC110535	3441	30	3.4	I	Skull B	Mandible frag.	1880−1630	Chamber
BETA593936	3430	30	3.2	I	Skull 3	Tooth	1875−1625	Corridor
SUERC110534	3412	30	3.3	I	Skull 4	Mandible frag.	1870−1620	Corridor
SUERC110540	3372	23	3.4	I	Skull 1	Mandible frag.	1740−1545	Corridor
BETA593937	3370	30	3.2	I	Individual	Tooth	1740−1540	Chamber
SUERC110531	3343	28	3.4	I	Skull 5	Mandible frag.	1735−1535	Corridor
SUERC110530	3248	28	3.4	I	Skull 2	Mandible frag.	1610−1440	Corridor
BETA633775	3510	30	3.2	II	Bone assemblage 4	Tooth	1920−1745	Chamber
BETA633777	3480	30	3.2	II	Bone assemblage 5	Tooth	1890−1695	Chamber
BETA633773	3440	30	3.3	II	Bone assemblage 2	Tooth	1880−1630	Chamber
BETA633774	3420	30	3.2	II	Bone assemblage 3	Tooth	1875−1620	Chamber
BETA633772	3380	30	3.2	II	Bone assemblage 1	Tooth	1745−1545	Corridor
BETA633776	3360	30	3.3	II	Bone assemblage 8	Tooth	1740−1540	Chamber

In both cases, given the secondary and collective nature of the burials, samples were taken from mandible fragments and teeth samples in order to allow individualization. The selection of samples for radiocarbon dating also aimed to ensure a balanced representation of the spatial distribution within each tomb distinguishing between remains from the chambers and corridors of both tombs, as well as including the adult female individual in a primary position in Tomb I and bone assemblages 1, 2, 3, 4, 5, and 8 from Tomb II.

The extracted bone collagen showed good preservation (see Table 1), with %C values ranging from 37.83% to 42.8% and %N values from 13.0% to 15.42%. All samples present acceptable C/N ratios ranging between 3.2 and 3.5 (mean = 3.3).

The *δ^13^*C and δ^15^N isotope values from Tomb I range from –19.5‰ to –18.8‰ for *δ1*C (mean: –19.1‰) and from 7.9‰ to 9.8‰ for *δ*^15^N (mean: 8.93‰). In Tomb II, values range from −20.0 to −18.3‰ for^13^C (mean: –19.1‰), while *δ*^15^N values range from 7.8‰ to 10.7‰ for (mean: 9.45‰) (S1 Table and S1 File). These results suggest limited marine input and therefore a minimal reservoir effect. All radiocarbon dates were calibrated using *OxCal v4.4* [30] with the *IntCal20* calibration curve [31].

Subsequently, the new set of radiocarbon dates was analysed using different Bayesian models [30] and the Chi-Square test [32], both implemented in *OxCal v4.4*. These analyses aimed to: 1) to determine the chronological framework of the two tombs and assess their degree of contemporaneity, and 2) to establish the temporal relationships among the individuals who were dated, both within and between the tombs.

### 3.4. Archaeometric studies: Grave goods

Tomb I yielded the following funerary goods: two gold earrings; three silver beads (two spherical and one tubular); three green mica beads; an ivory comb; a disc-shaped shell; and a perforated shell.

Tomb II presented fewer funerary goods: an arsenic-rich copper ring; an arsenic-rich copper arrowhead; and a necklace bead (green mica).

Due to the singularity and fragility of the archaeological objects, non-destructive techniques (NDT) were employed to preserve their integrity. Accordingly, the used methods included: (1) stereoscopic microscopy (SM), using a Nikon SZ microscope (magnifications ranging from x30 to x60); (2) Environmental Scanning Electron Microscopy (ESEM) SEI-Nano SEM microscope (magnifications from x30 to x5000), equipped with elemental chemical microanalysis EDX; (3) X-ray diffraction (XRD), performed with a Bruker D-8 Advance system under the following conditions: Cu Kα radiation, and in specific cases using direct diffraction with a Göbel mirror, since the samples were not analysed in powder form but rather through direct examination of the unaltered surfaces of the objects; (4) micro-X-ray Fluorescence Spectroscopy (XRF), employing Rh radiation; (5) Raman spectroscopy, conducted with a Raman microscope BWTek equipped with a 785 nm laser; and finally, (6) FTIR spectroscopy was performed on the ivory comb using a spectrometer Alpha by Bruker. All analyses were carried out in the laboratories of SCICYT and the Geoarchaeology and Archaeometry Unit at the University of Cádiz (UGEA-PHAM, UCA).

### 3.5. Ceramic studies

The assemblage also comprises 687 ceramic fragments, of which 434 belong to Tomb I and 253 to Tomb II. The quantification methodology used different variables, such as fragment frequency (NFR) and the number of rims, to establish the minimum number of individuals (MNI) and weight (gr.) and density in stratigraphic contexts [33,34]. Technological and surface treatment analysis was undertaken with a macroscopic binocular loupe. Petrographic characterisation, among other features, will require the use of other techniques in future studies.

A typological proposal was made with the fragments that allowed the reconstruction of reliable profiles. The groups follow morphological criteria set out by earlier studies of Bronze Age pottery in the south of the Iberian peninsula [35,36].

## 4. Results

### 4.1. Architectural analysis

The digital records allowed for a thorough architectural analysis of the features. Base data and 3D scanner and photogrammetry recording protocols can be consulted in the Supplementary Material (S1 Fig and S2 Fig).

Tomb I: is a compound or mixed type tomb, with an orthostatic corridor and a hypogenic chamber (Fig 2a). It matches Type III b in Encarnación Rivero’s typology of artificial caves in Andalusia and Portugal [37]. The corridor is 2.57 m long from the entrance to the underground chamber, and from 0.46 m (access) to 0.71 m (final section) wide. On each side of the corridor there are four vertical orthostates, between 1 and 1.15 m high and 0.50 and 0.62 m wide. The corridor ceiling consists of large stone slabs arranged transversely, resting atop the vertical slabs. During the excavation, the cover had collapsed, and none of the slabs were on their original horizontal position (Fig 3).

**Fig 2:**
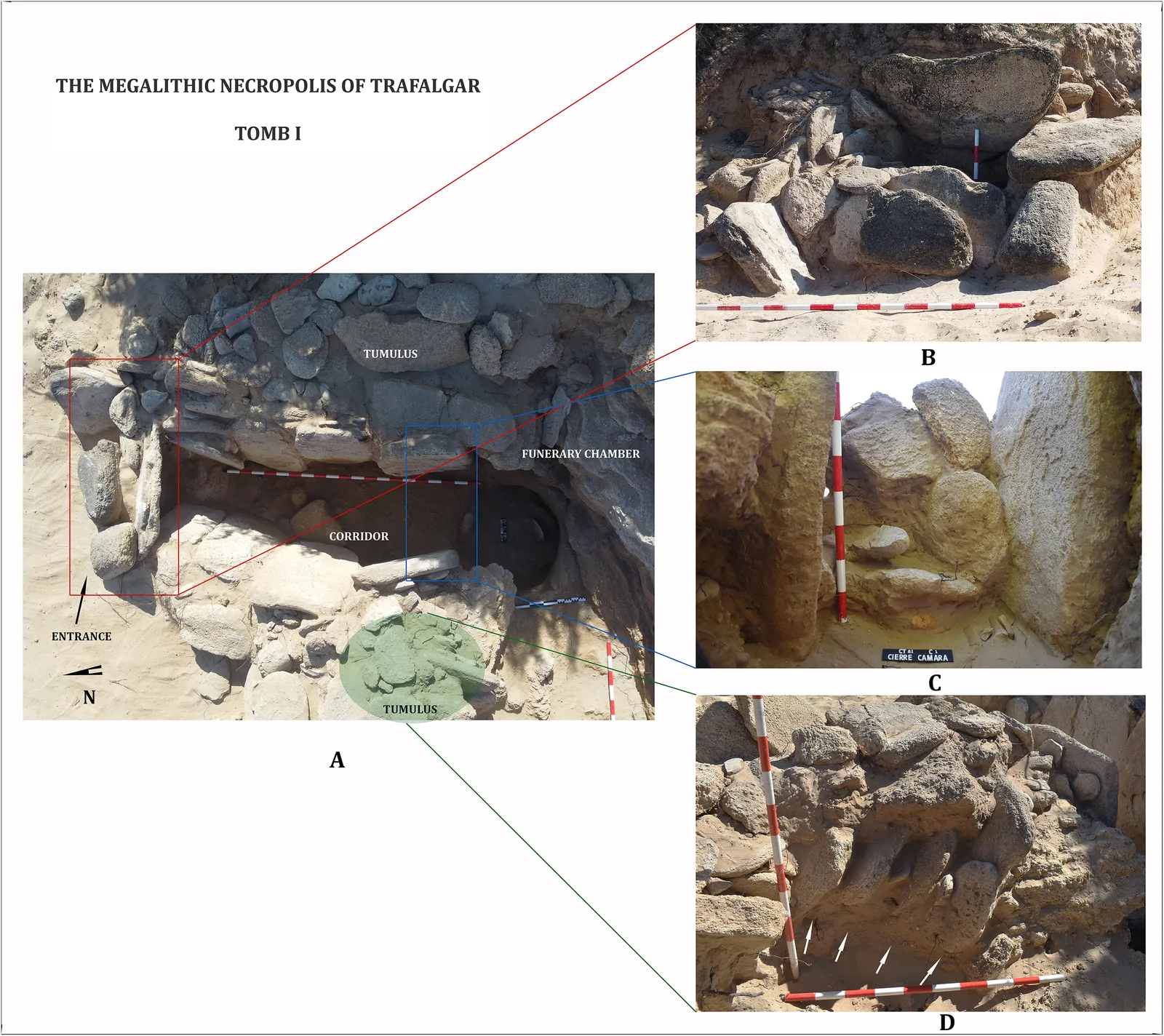
(A) Aerial view of Tomb I. (B) Accumulation of small stones that sealed the access to the corridor. (C) Accumulation of stones sealing the final part of the corridor. (D) Section of the tumulus left in reserve, comprising several layers of stones and sand.

**Fig 3:**
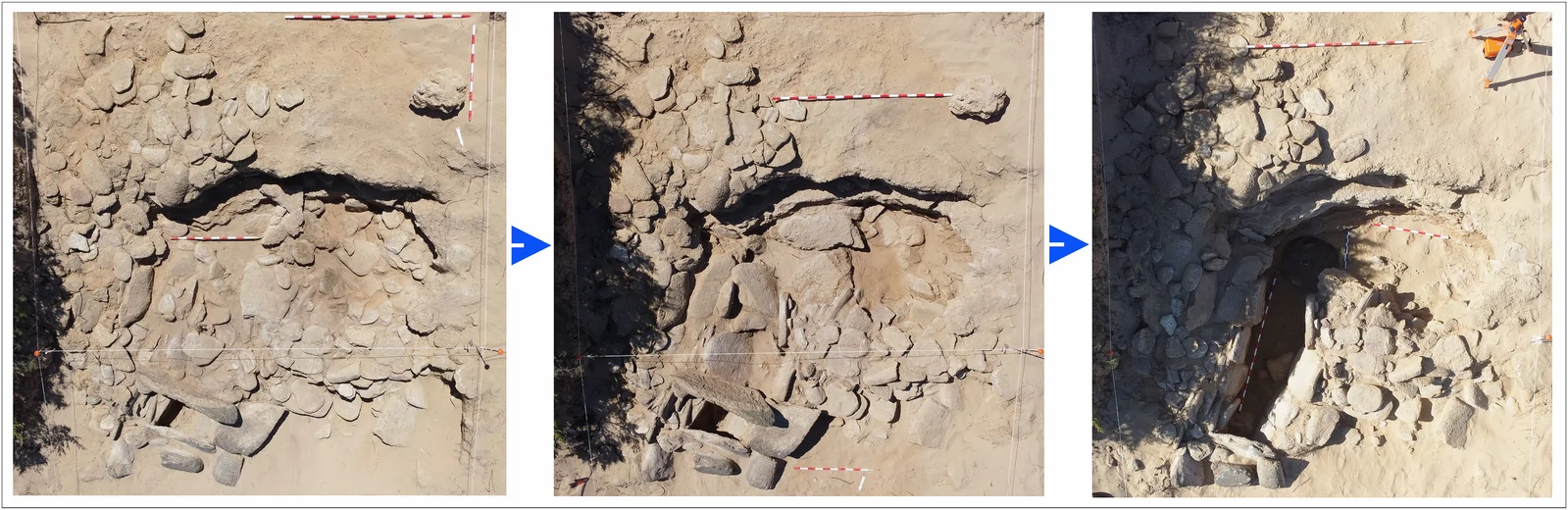
Aerial view of Tomb I during excavation: stone tumulus (left); collapsed slab covers (centre); and corridor and entrance of the chamber after the removal of the roofing (right).

The funerary chamber is excavated in the geological substratum. Its shape is ovale, with a maximum axis of 2.32 m and a smaller one of 1.29 m. The maximum height of the vaulted area, from bottom to top, is 1.37 m (Fig 4a). During the excavation, the presence of blocks detached from the walls was attested, which suggests that there was an aerial part that has collapsed, reducing the hypogeum to an underground chamber located below the corridor’s floor level. Inside, in the central area, the remains of an anatomically-connected adult individual was documented, as well as secondary bone sets in the areas close to the walls.

**Fig 4:**
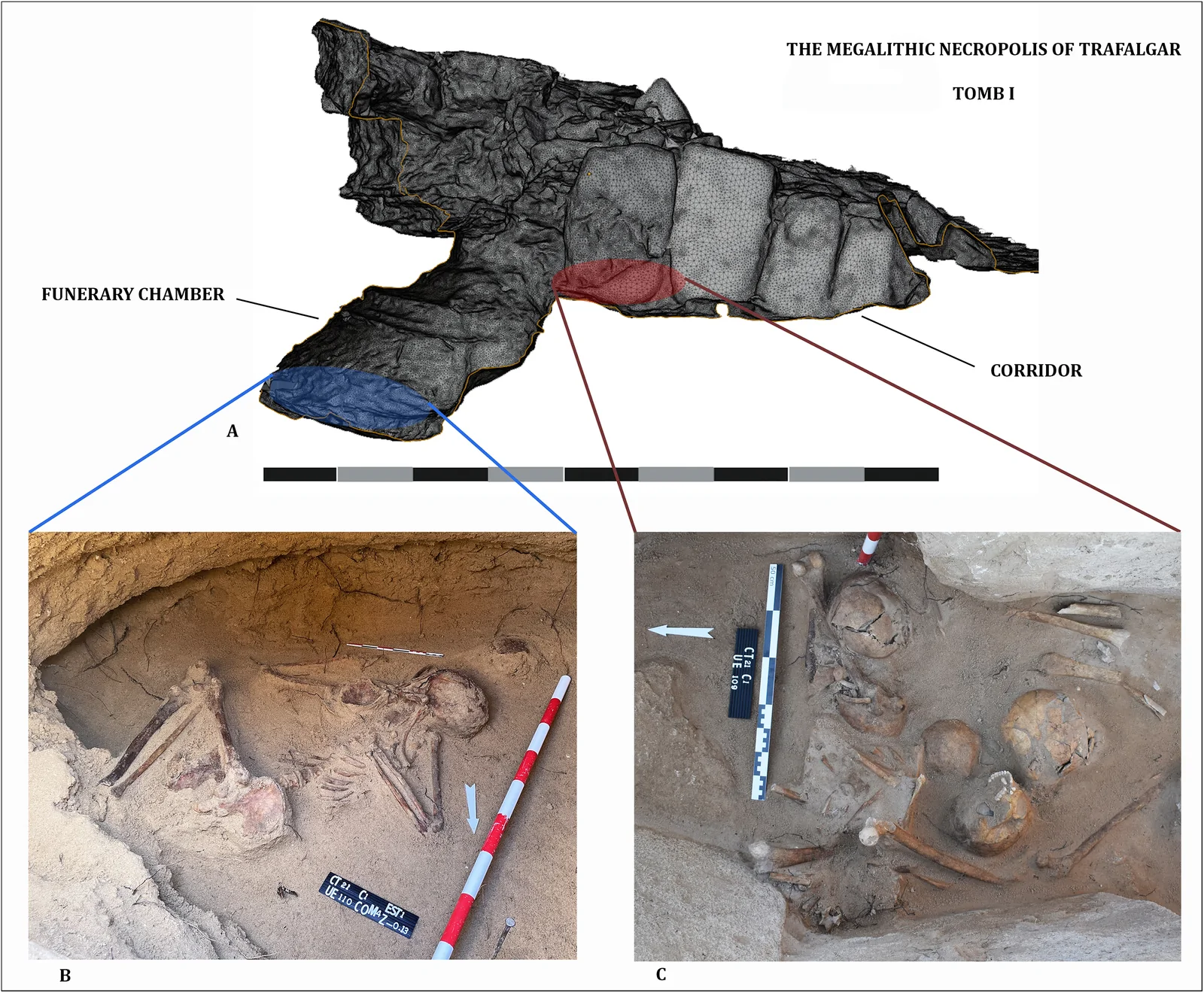
(A) Longitudinal section created using Leica 3DR software, showing the orthostatic corridor and the burial chamber excavated underground (hypogeum); (B) individual in anatomical connection found at the base of the burial chamber; (C) secondary bone set found at the end of the corridor under the accumulation of stones that sealed the entrance to the burial chamber.

The access to the tomb was double sealed at the entrance and final section of the corridor. The first seal consisted of a slab placed transversally to the first two orthostates, and a series of smaller stones on top (Fig 2b). The chamber entrance was blocked with a wall of medium and large stones, built upon an earlier bone deposit (Figs 2c and 4c).

The whole funerary structure was covered with a tumulus made of medium-size stone layers filled with sand (Fig 2d). The rocks of the tumulus lay on a fossil dune and part of the tombolo’s rocky front. The estimated diameter based on the dispersion of the stone slabs is approximately 4 m.

Tomb I is north-oriented, although with a slight east deviation, alignment defined by the axis of the corridor. This inland-facing orientation seems to be determined by the disposition of the rocky front where the hypogenic chamber is cut.

Tomb II: The tholos-type Tomb II (Fig 1) is at a distance of 180 m from Tomb I. It is oriented to the southeast and, unlike Tomb I, has an ample view of the horizon and the sea. It includes a small corridor that leads to a circular chamber. The corridor, 2.32 m long and 1.02 m wide, has a rectangular plan with eight orthostates, four on each side, with heights ranging from 1 to 1.15 m. Over the orthostates, three horizontal slabs form a lintelled ceiling. One of these was found in its original position, another one had collapsed, and one was displaced (Fig 5). Access to the chamber was blocked by a slab sitting on a bed of medium-sized stones wedged in by smaller ones (Fig 6c and 6d). This slab played a key role in securing the only covering slab preserved in situ (Fig 6c). The access corridor was sealed by two vertical slabs and a stone plug (Fig 6a). In the corridor, a bone assemblage found at a higher elevation than the original floor level was documented, sitting over a packet of dune sand, which suggests that this deposit was done after the tomb was sealed and abandoned, probably by lifting one of the covering slabs of the corridor (Fig 8a and 8c).

**Fig 5:**
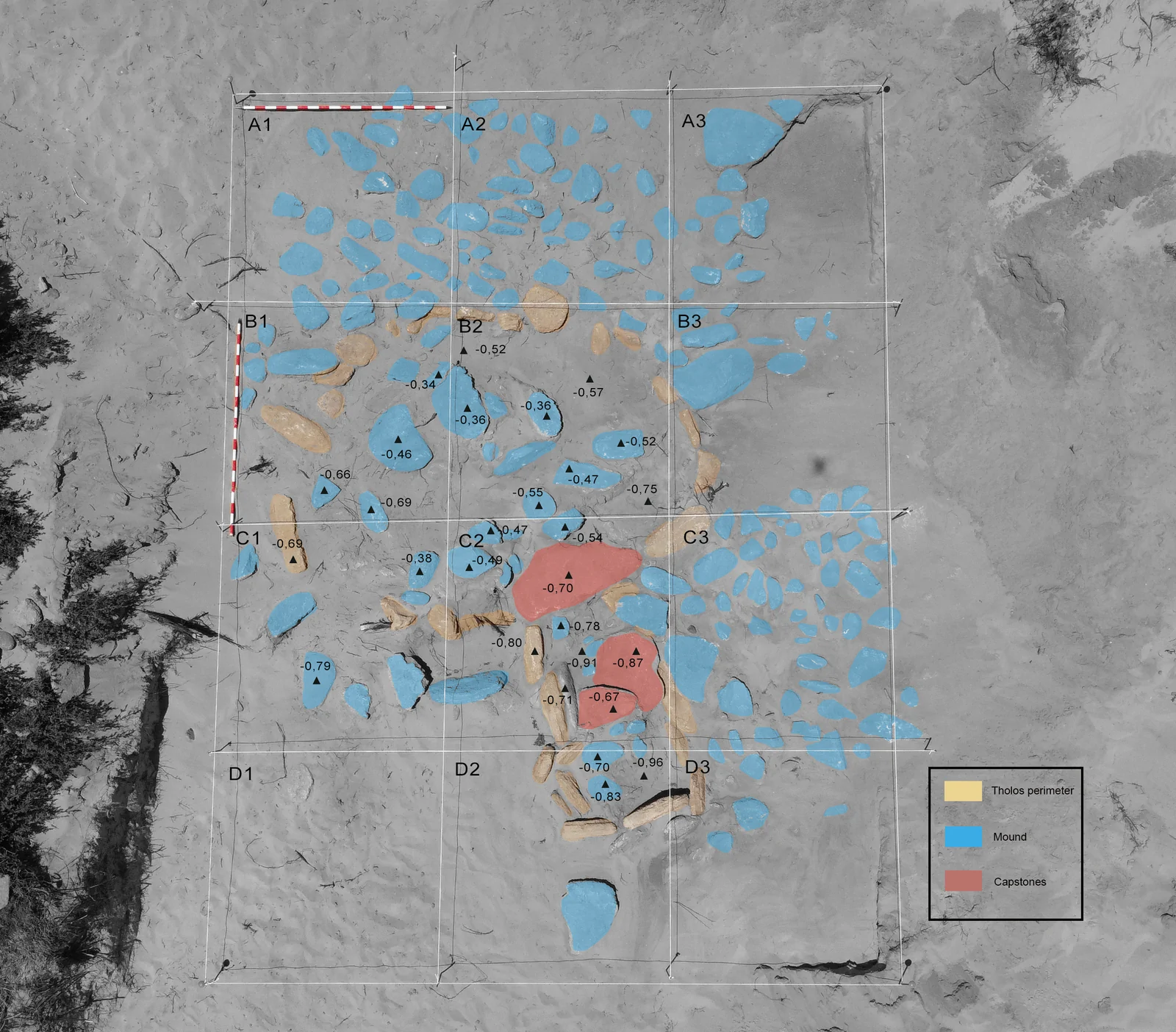
Initial floor of Tomb II after the removal of the dune that covered it. The perimeter (in yellow), the stones that form the tumulus (blue), and the ridge tiles preserved in the corridor (red) can be observed.

**Fig 6:**
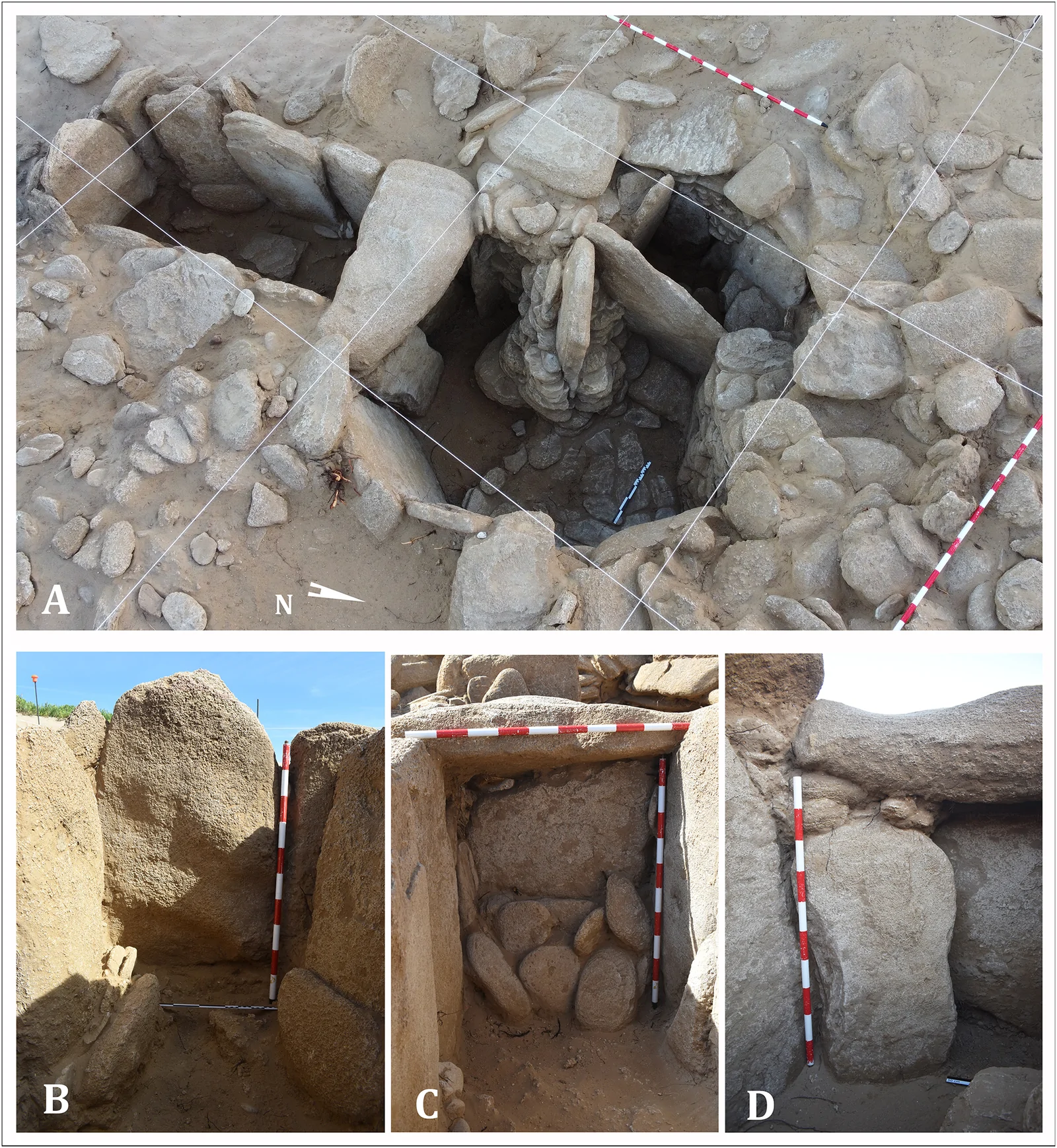
(A) Aerial view of Tomb II; (B) orthostate that sealed the corridor entrance; (C and D) orthostate located at the end of the corridor sealing the access to the burial chamber.

The corridor gives access to a circular funerary chamber with a maximum diameter of 2.90 m. The maximum height of the chamber, from the floor to the highest part of the ceiling, is of 1.5 m. The chamber’s walls are made with ten sandstone/conglomerate orthostates. The irregular morphology of these vertical slabs, which vary in height and shape, pushed the builders to reinforce the resulting gaps with masonry. This mixed constructive technique gave consistency to the structure, which was also reinforced with small angular stones wedged into the base of the orthostates.

One of this tomb’s most peculiar architectural features is the presence of a pillar in the chamber’s central area, formed by three vertical orthostates, set in 90º and 45º angles on a masonry plinth (Fig 7a, 7b and 7c). A masonry wall attached to the chamber’s northern orthostatic wall was also attested (Fig 7d). These reinforcing elements suggest that the original false dome of the burial chamber must have collapsed some time before the monument was closed. This must have required the remodelling of the funerary chamber; the construction of the central pillar and the filling of the western half of the structure added structural stability by significantly reducing the chamber’s surface. The Leica 3DR software was used to verify this reduction in usable space from 6.54 m^2^ to 2.65 m^2^, that is, to just over 1/3 of the initial area. As part of this remodelling process, a stone pavement, where the skeletal remains were to be deposited, was laid between pillars 1 and 2 (Fig 8 and Fig 7b). These repairs appear to have been successful, since the tomb was found full of a sandy sediment filtered from the dunes outside, over which the vaulted roof finally collapsed. The roof was a false vault; the finds include a deposit of stone slabs inside the chamber, the collapsed ceiling, and three courses of masonry still *in situ* projecting towards the centre to form the false vault. These courses rested on large horizontal slabs that outlined the external perimeter. These stone features were laid out to form a uniform surface over which to raise the vaulted roof (Fig 9).

**Fig 7:**
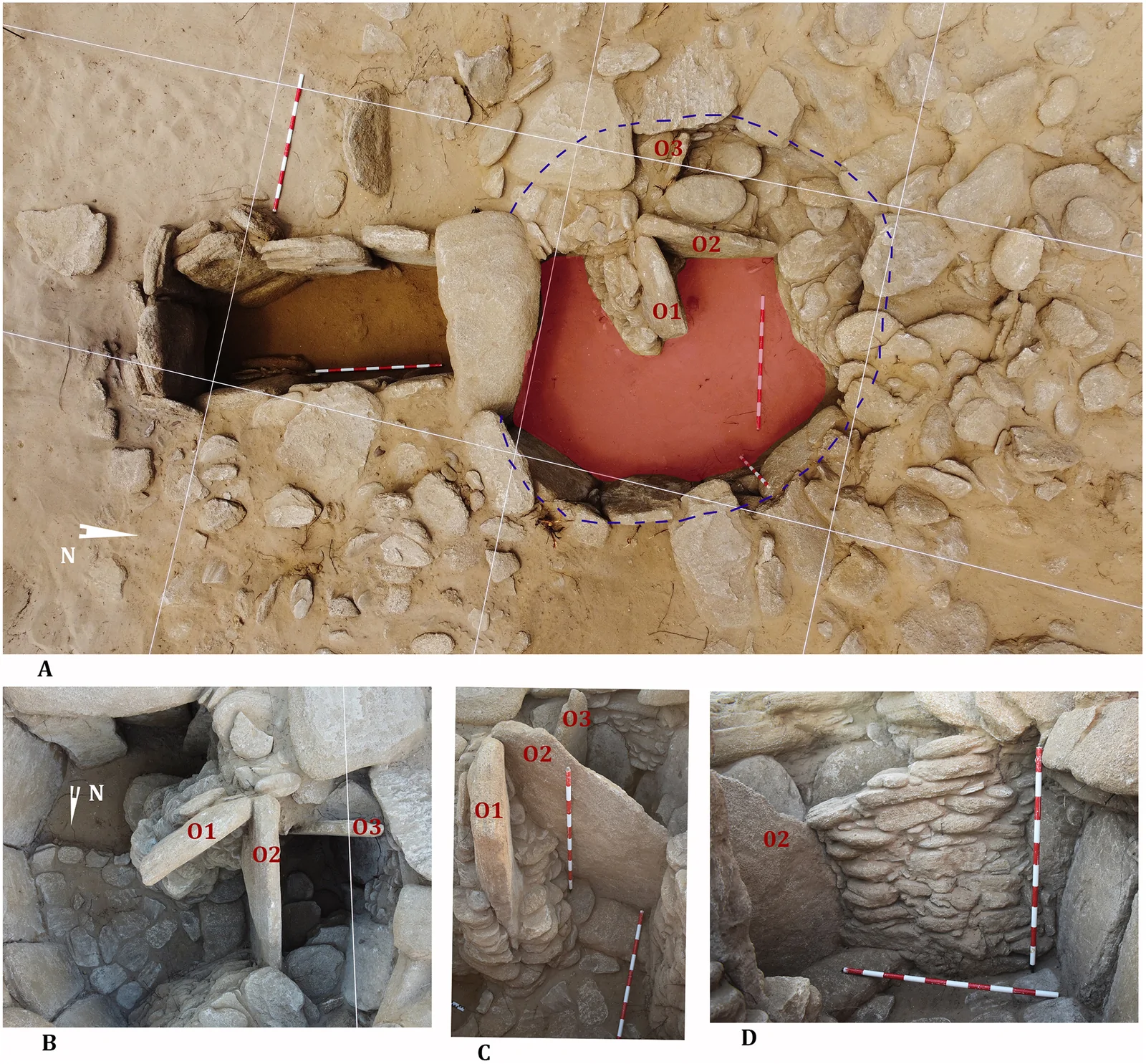
(A) Aerial view of Tomb II with an indication of the size of the original burial chamber (blue discontinuous line) and surface of the burial chamber after the remodelling (red); (B) aerial view of the pillar in burial chamber, formed by three orthostates; (C) feature made of small stones to reinforce the orthostates; (D) feature that seals off part of the western half of the burial chamber.

**Fig 8:**
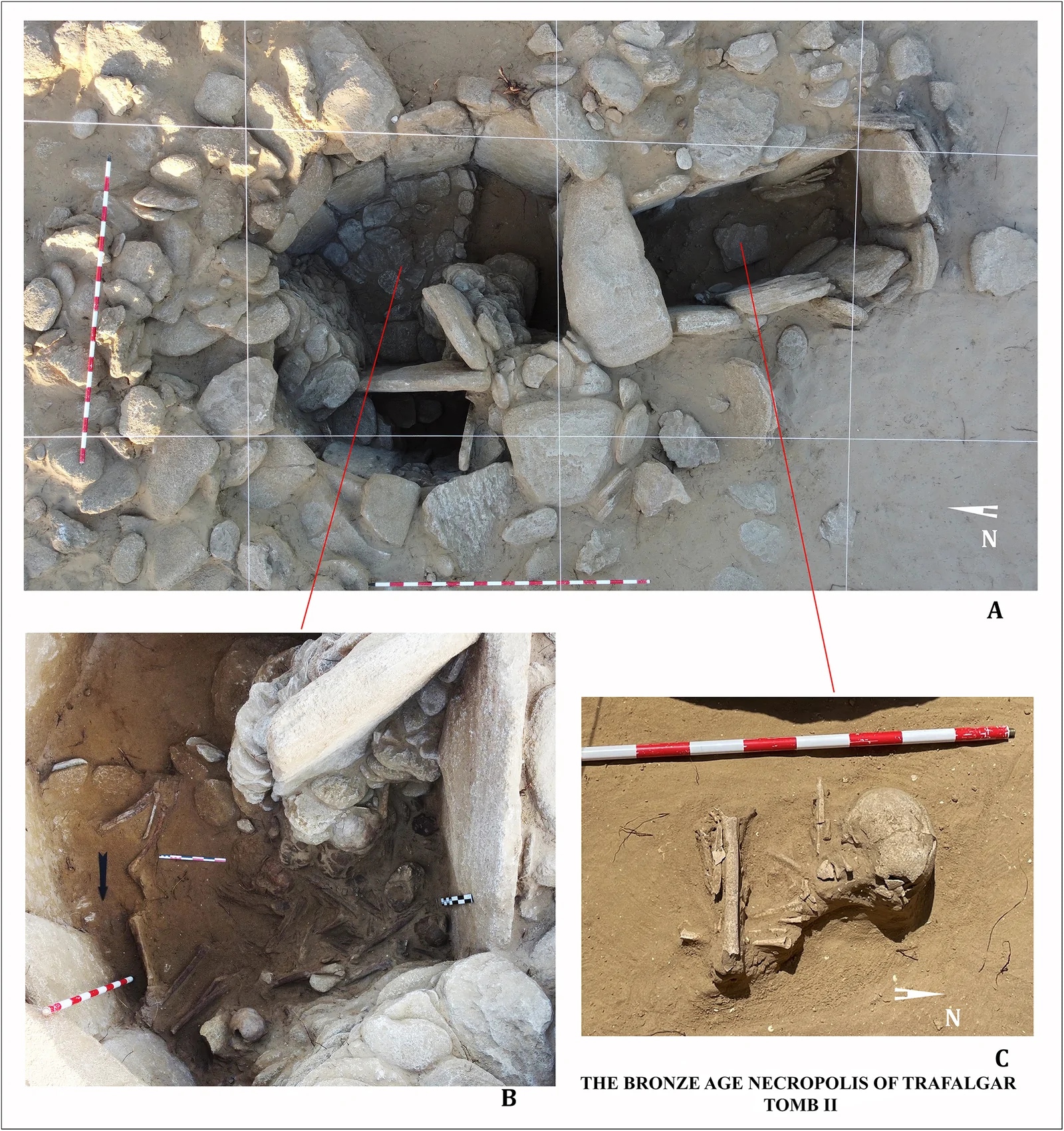
(A) Aerial view of Tomb II; (B) ossuary on the floor of the burial chamber; (C) partially articulated individual found in the corridor.

**Fig 9:**
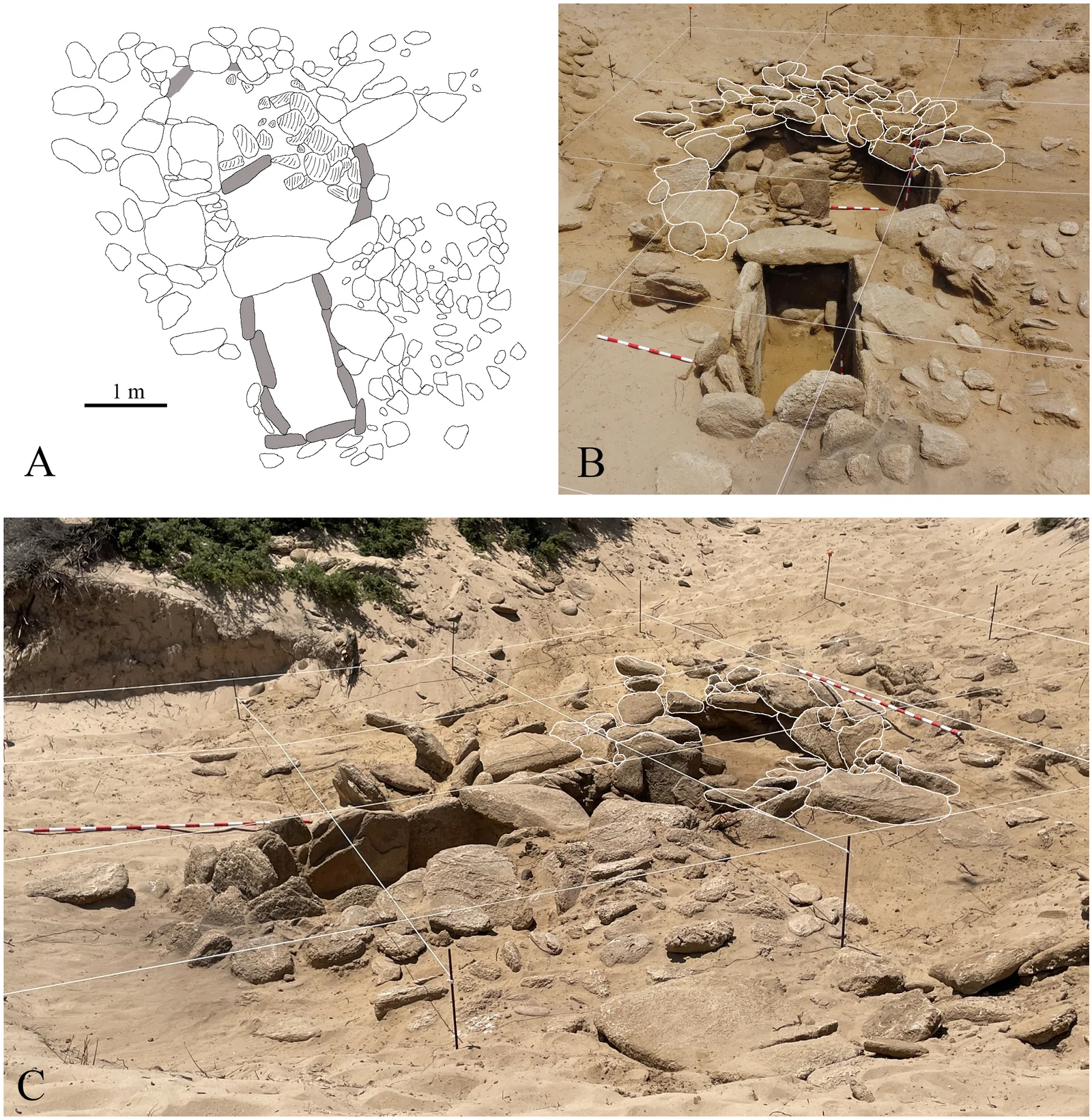
(A) Plan of Tomb II indicating the orthostats that comprise the corridor and the burial chamber (dark grey) and the remains of the collapsed dome (hatching). (B) Aerial view showing the three courses of masonry still *in situ* projecting towards the centre to form the false vault. (C) Detail of the three courses of masonry *in situ* that formed the false dome and the large horizontal slabs arranged around the perimeter of the burial chamber.

The raw building materials used in the construction of both structures were sourced from the platform conglomerates and the bioclastic sandstones of the Upper Pleistocene fossil dunes, which are located in tombolo of Trafalgar.

Finally, renders of both graves were generated for educational use, providing an immersive experience for all audiences, as illustrated in the exhibitions held in the museums of Cádiz and Vejer de la Frontera’s in 2022 [38]. In addition, these 3D models enabled the creation of highly accurate resin mock-ups as a museographic resource for the exhibit “El Megalitismo en las tierras de Cádiz” held in Conjunto Arqueológico de los Dólmenes de Antequera in 2024–2025 [39]. The value of new digital recording technologies for the preservation and value of cultural heritage cannot be overemphasised.

### 4.2. Bioarchaeology

We conducted a detailed osteological inventory of the human remains from Cape Trafalgar, identifying 869 bones and teeth in Tomb I and 212 in Tomb II. The MNI was estimated using the highest Minimum Number of Elements (MNE), following Knüsel & Outram [19] and carrying out refitting exercises to ensure an accurate MNE estimation [23]. In Tomb I, the MNE of 13 was established based on mandibles, right scapulae, and upper right canines (FDI 13), confirming an MNI of 13. In Tomb II, the highest MNE was 7, based on the left zygomatic process, confirming an MNI of 7.

Sex and age estimations in Tomb I were based on five skulls, three mandibles, one cranium, and the skull and pelvis of the individual in anatomical connection buried in the chamber. In Tomb II, they were estimated based on two skulls, a mandible, and an isolated third molar from a juvenile [21]. Age-at-death was recorded in broad ranges [20] (Table 2).

**Table 2:** Sex and age identifications of individuals from Tombs I and II.

Structure	Individual	Element assessed	Sex	Age
Tomb 1	Skull 1	Skull	F	Y.Adult
Tomb 1	Skull 3	Skull	F	Juvenile
Tomb 1	Skull A	Skull	F	Indet.
Tomb 1	Isolated mandible	Mandible	M?	Indet.
Tomb 1	Skull 5	Skull	I	Child
Tomb 1	Skull 4	Skull	F?	Y.Adult
Tomb 1	Skull 2	Mandible	M?	Y.Adult
Tomb I	Skull B	Skull	F	M.Adult
Tomb I	Isolated mandible	Mandible	I	M.Adult
Tomb I	Individual in the chamber	Skull and pelvis	F	Y.Adult
Tomb II	Isolated tooth	28 (3rd molar)	I	Adolescent
Tomb II	Skull 1	Mandible	F?	Adult
Tomb II	Skull 5	Skull	M	Adult
Tomb II	Skull 4	Skull	F	Adult

In Tomb I, the funerary chamber was used first (1875−1700 cal BC), and an MNI of eight individuals have been documented, although only one was in anatomical position (radiocarbon dating indicates that this was the last burial before the chamber was closed for funerary use). Furthermore, this female individual was buried with prestigious grave goods, including two gold earrings, three necklace silver beads, an ivory comb, and several green mica beads. At the end of the corridor, there was a collective burial deposit with an NMI of five individuals, under a pile of stones that sealed the end of the corridor (Fig 2C). This collective deposit is more recent: 1740−1630 cal BC. In Tomb II, the earliest phase concerns the burial chamber (1875−1700 cal BC), with a mixed collective burial (MNI = 6 individuals) (Fig 8b) and a more recent phase (1740−1630 cal BC), including the individual in partial anatomical connection, found in the corridor, and one of the individuals in the burial chamber (Fig 8c). By the time the individual in the corridor was buried, this space had silted up significantly. An arsenic-rich copper earring was found next to this individual, making it the only grave offering found in association with a burial in this tomb, since the arsenic-rich copper arrowhead was found in the funerary tumulus.

### 4.3. Radiocarbon dates and Bayesian analysis

A Single Contiguous Phase Bayesian model indicated a continuous temporal distribution of both tombs (Fig 10, Table 3.). Tomb I is dated between 1925–1710 and 1735–1540 cal BC, with an estimated time span of 15–255 years (*Amodel* = 89.6; *Aoverall* = 85.6), while Tomb II ranges from 1990–1705–1740–1485 cal BC, spanning 8–254 years (*Amodel* = 98.1; *Aoverall* = 96.6). The model identified one outlier, SUERC-10530 date, associated with Skull 2 in the corridor of Tomb I, which yielded a slightly later date of 1610–1440 cal BC.

**Table 3:** Single Contiguous Phase Bayesian Model of the radiocarbon dates of Tomb I and Tomb II.

	Boundary Start (95.4% probability)	Boundary End (95.4% probability)	Span (95.4% probability)	Agreement indices
TOMB I	1920−1710	1735−1540	15-255	Amodel = 89.6 Aoverall = 85.6
TOMB II	1975−1705	1740−1500	0-250	Amodel = 96.5 Aoverall = 94.5

**Fig 10:**
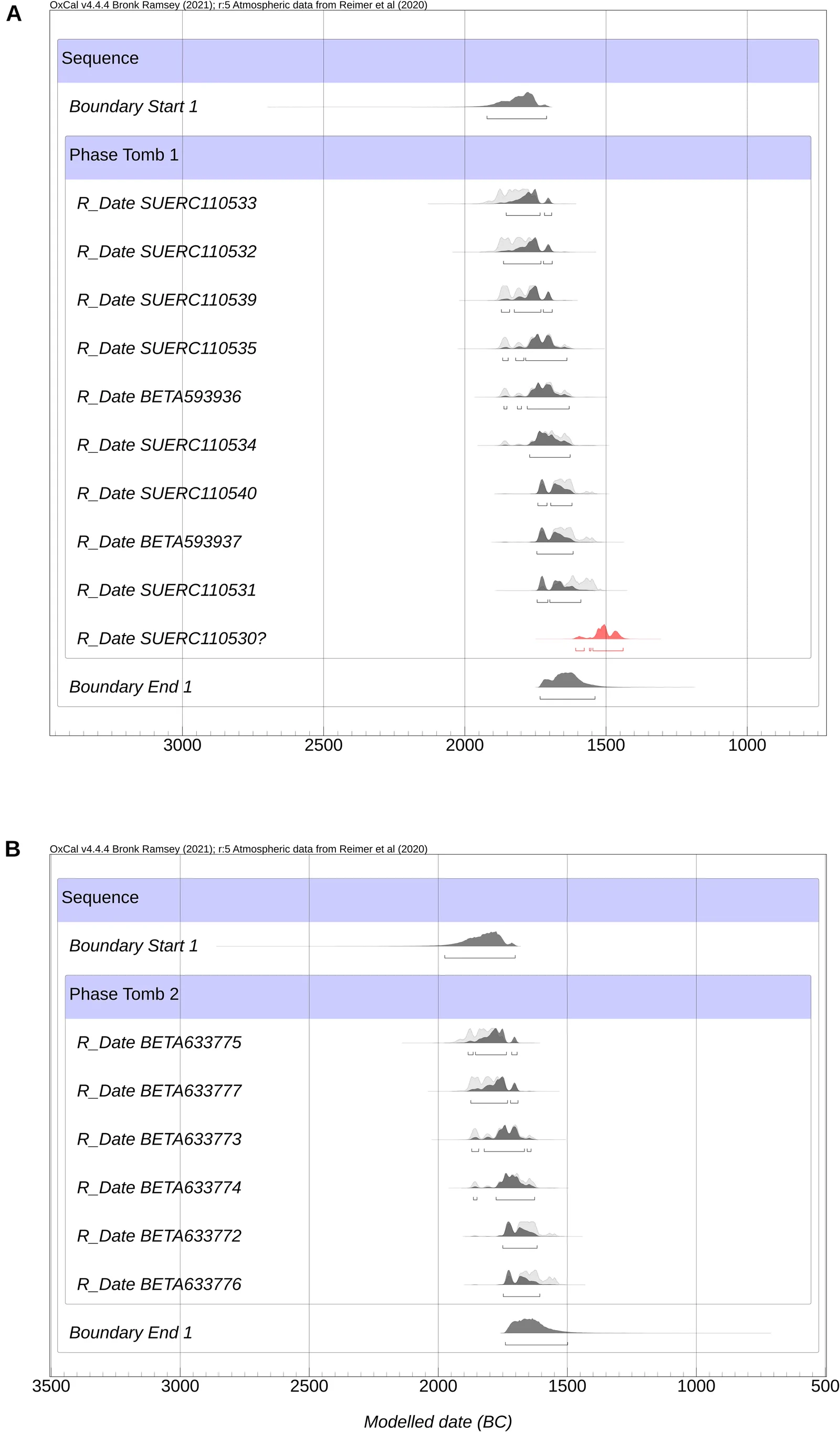
Single contiguous phase bayesian model of the radiocarbon dates of Tomb I (left) and Tomb II (right). Agreement and convergence indices are shown for each determination: A = individual agreement index; C = convergence index. Amodel indicates the overall agreement index of the model. The outlier (SUERC-10530) is highlighted in red and the value P corresponds to the posterior outlier probability.

To further explore the internal temporal distribution of funerary activity within each tomb, Kernel Density Estimation (KDE) models were constructed using OxCal. Unlike the Bayesian phase model, which estimates the boundaries and duration of use, the KDE provides a smoothed representation of the density of dated events through time.

The KDE model of Tomb I (Fig 11) reveals a unimodal distribution with a clear peak centred around 1750–1700 cal BC, indicating the period of highest funerary activity (Amodel 84.1 and Aoverall 83.6). The distribution extends from approximately 1900–1500 cal BC, although most dates cluster within a narrower interval between 1850 and 1650 cal BC. Taken together, the Single Contiguous Phase and KDE models suggest a single, intense temporal dynamic of funerary activity in the necropolis. Excluding the isolated later determination SUERC-110530, associated with Cranium 2 in the corridor of Tomb I, the dated depositions fall within a relatively short interval, broadly between ca. 1875 and 1600 cal BC. This pattern support a main phase of funerary use, followed by an isolated later episode of deposition or reuse in the corridor of Tomb I, dated to 1610–1440 cal BC.

**Fig 11:**
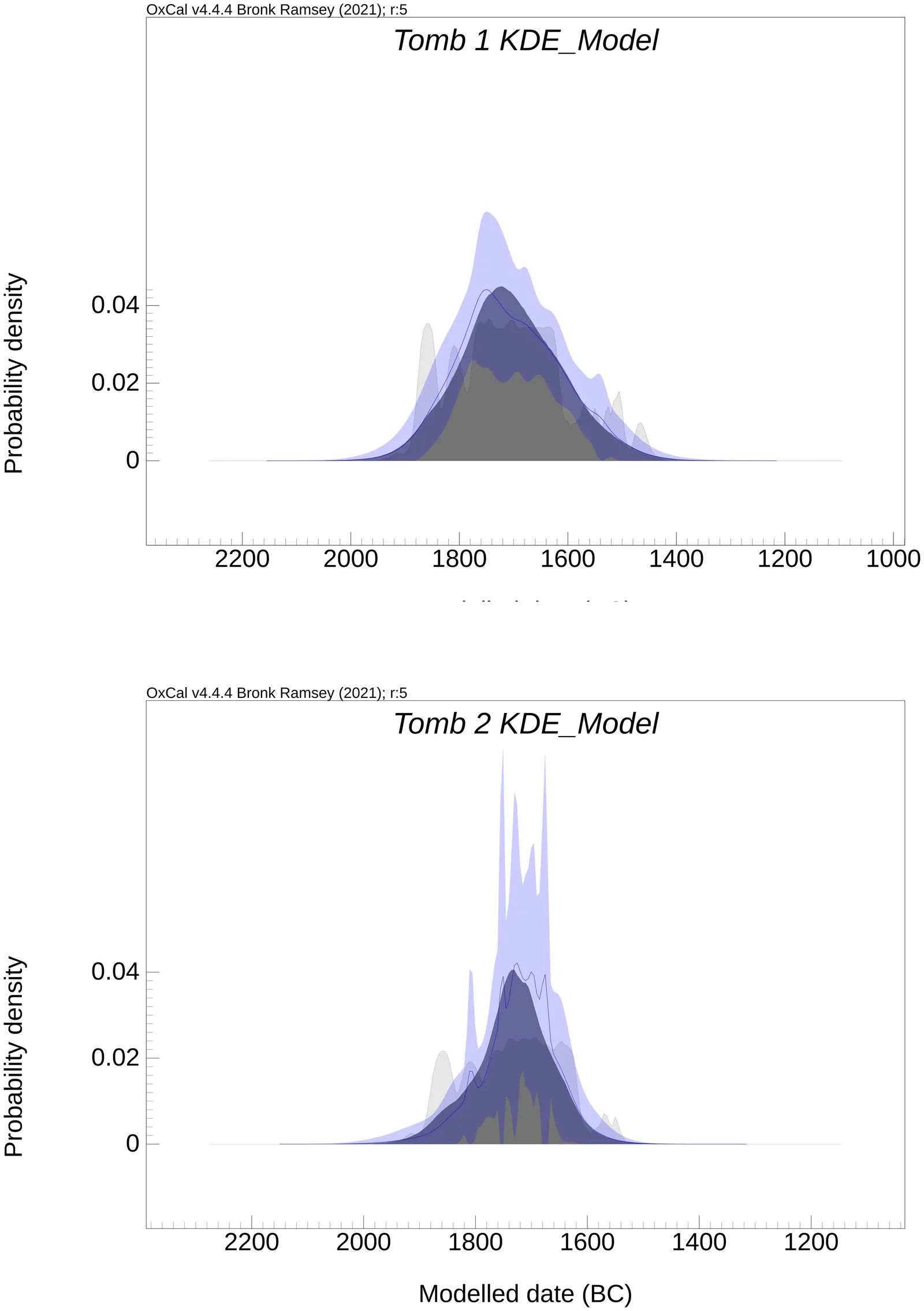
KDE Model of Tomb I (left) and Tomb II (right) radiocarbon dates.

The KDE model for Tomb II (Fig 11) shows a narrow and well-defined unimodal distribution centred around 1750–1680 cal BC, indicating a temporally concentrated phase of funerary activity. In contrast to Tomb I, the distribution lacks a significant late tail, suggesting the absence of substantial later reuse. While both tombs share a common period of maximum activity, Tomb II exhibits a more constrained temporal pattern, whereas Tomb I displays a broader distribution extending into later periods. This difference suggests distinct temporal dynamics in the use of the two funerary structures despite their overall contemporaneity.

The chi-square tests of radiocarbon dates from Tombs I and II reveal distinct temporal patterns in relation to both burial chronology and spatial organization within the tombs (Fig 12 and 13). In Tomb I**,** three partially overlapping chronological clusters can be observed: an early group of inhumations broadly dated to 1875–1740 cal BC, a middle group dated to 1740–1630 cal BC, both mainly concentrated in the chamber**,** and a later partially overlapping group dated to ca. 1735–1625 cal BC, primarily associated with the corridor**.** This spatial-temporal pattern suggests a sequential use of the tomb, with the chamber being used first and the corridor subsecuently in later phases. These clusters should therefore be understood as short episodes of death and deposition within a broader single phase of funerary activity, probably spanning only a few generations. In this sense, the chi-square results suggest a gradual shift in funerary activity from the chamber towards the corridor, rather than cleary separated phases or a pattern of prolonged, recurrent reuse. Notably, the latest date from the chamber belongs to the only individual (an adult female) found in primary position, indicating that this formal, intentional deposition may have marked a significant event or transition in the use of the tomb. A single outlier (SUERC110530) from the corridor appears significantly later (1610–1440 cal BC), potentially representing a re-use or secondary deposition. Thus, chronologically, most of the dated individuals at Tomb I can be ascribed to the Middle Bronze Age with Skull 2 dating towards the end of this period. In contrast, Tomb II shows a more compressed and homogeneous chronological framework, with two main contemporary clusters: one between 1875–1700 cal BC, mostly located in the chamber, and another one between 1740–1630 cal BC, which includes both chamber and corridor burials. These patterns point to a more staggered and possibly extended use of Tomb I compared to the more temporally concentrated use of Tomb II.

**Fig 12:**
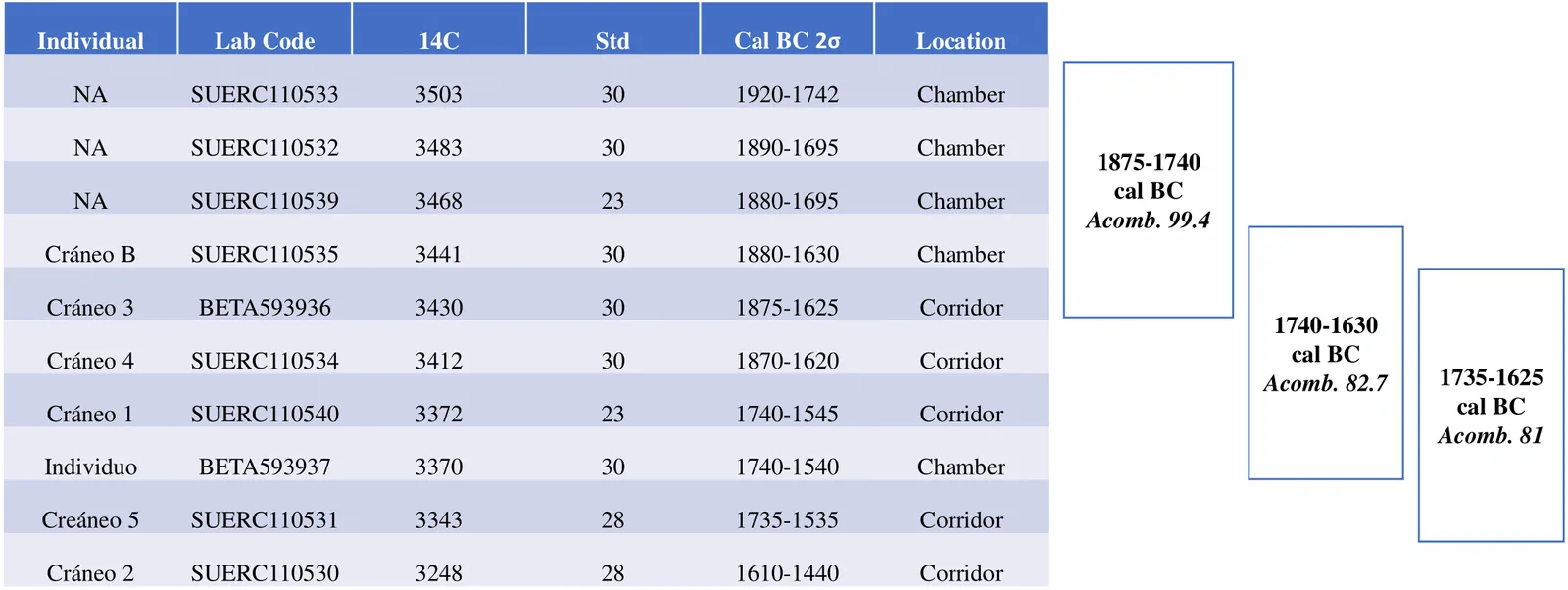
Contemporary groups in Tomb I according to the chi-square test of radiocarbon dates. Values shown correspond to OxCal Combine agreement indices (Acomb) resulting from the chi-square test; values above 60% indicate acceptable agreement among the combined determinations.

**Fig 13:**
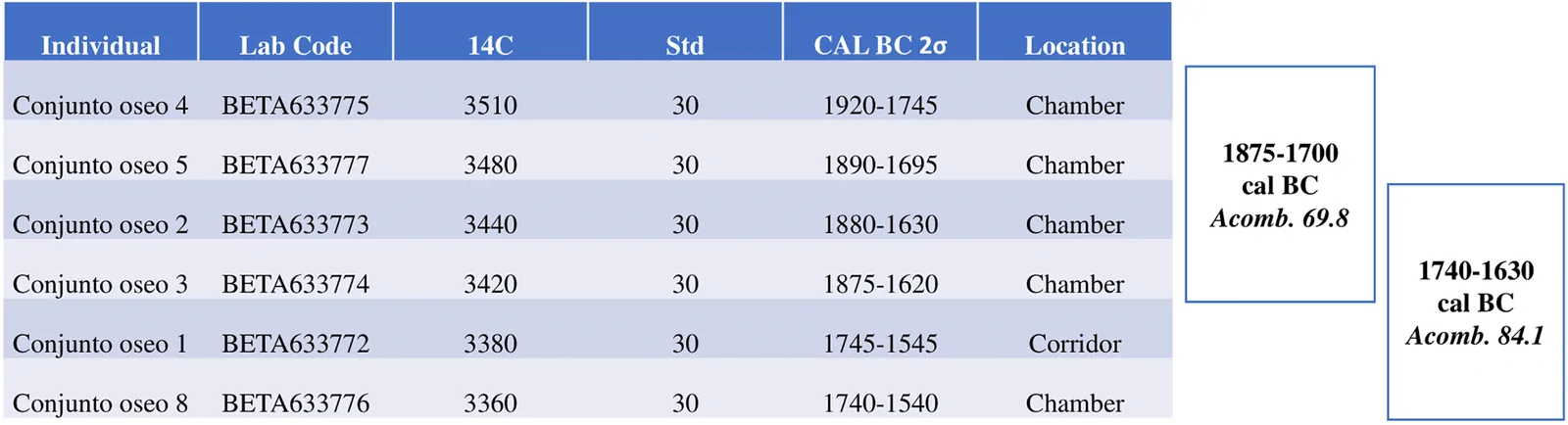
Contemporary groups in Tomb II according to the chi-square test of radiocarbon dates. Acomb = Agreement indices.

The combined chi-square analysis of all radiocarbon dates from Tombs I and II reveals a pattern of overlapping funerary use, indicating that both tombs were simultaneously in use during multiple moments (Fig 14). The results identify at least four chronological clusters, each of one includes individuals from both tombs, reinforcing the interpretation of no temporal discontinuity in their use —any tomb was active while the other was not. The earlier radiocarbon dates (1875–1700 cal BC) are dominated by inhumations in the chambers, while the later ones (1740–1630 cal BC) increasingly involve the corridors**,** particularly in Tomb I. This spatial shift may reflect changes in funerary practices or spatial constraints over time.

**Fig 14:**
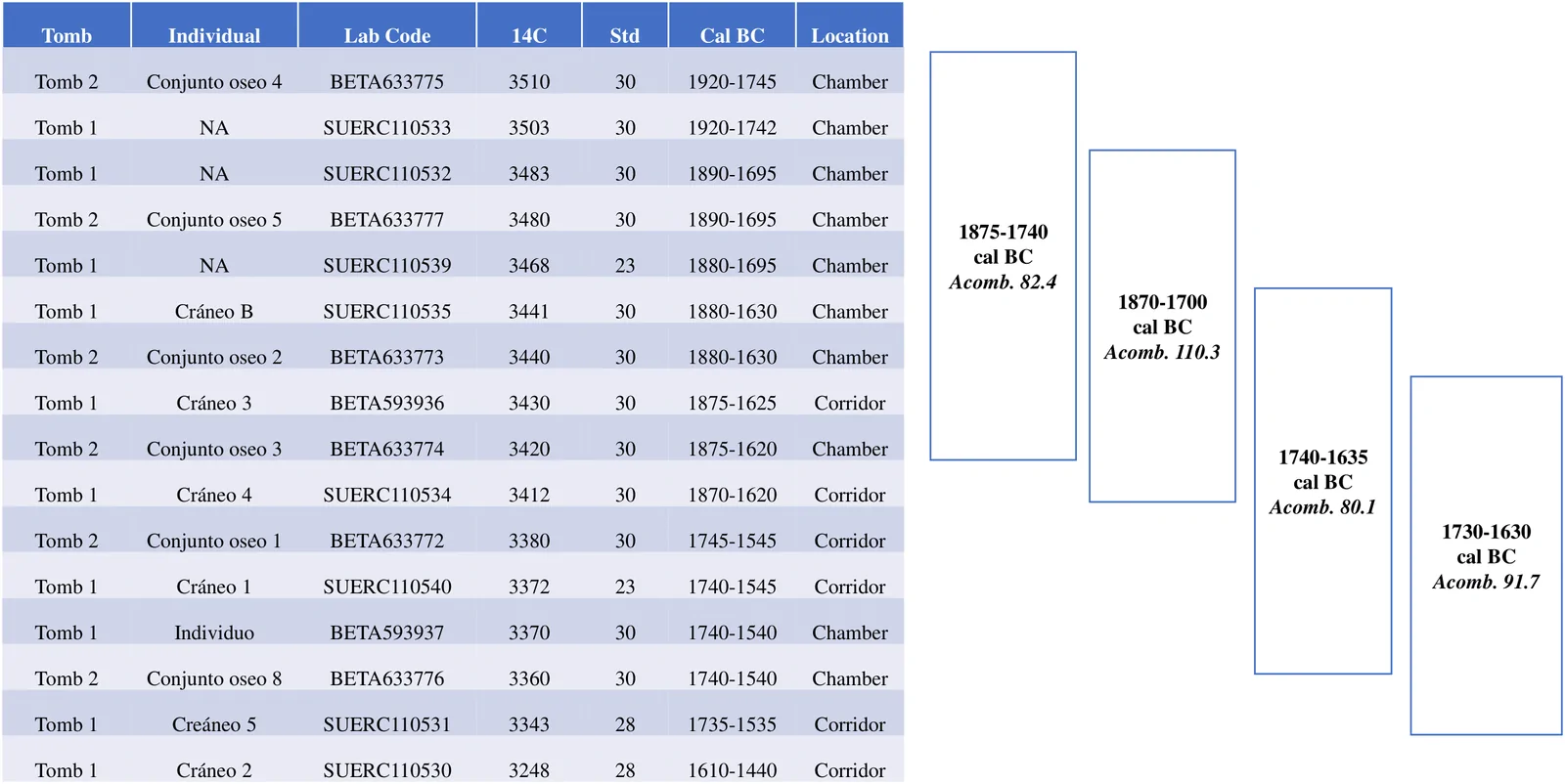
Contemporary groups in Tomb I and Tomb II according to the chi-square test of radiocarbon dates. Acomb = Agreement indices.

While Tomb I shows evidence of a slightly longer and more diachronic use—including notably late deposition of on the corridor (Individual skull 2, outlier in the previous Single Contiguous Phase Bayesian Model). In contrast, Tomb II presents a more internally consistent pattern, yet still aligns chronologically with the timeline use of Tomb I. Overall, these results support the interpretation of both tombs as contemporaneous and functionally connected funerary spaces, probably used within a relatively short and intense period of funerary activity. The chronological clusters identified by the chi-square tests therefore appear to reflect the internal tempo and spatial organization of burial practices within a single funerary cycle, rather than distinct phases of long-term reuse.

Together, the Bayesian modelling, KDE analysis, and chi-square clustering provide a multi-scalar chronological framework, demonstrating both the contemporaneous use of the two tombs and subtle differences in their internal temporal dynamics.

### 4.4. Grave goods

The grave goods recovered from the two tombs excavated at the tombolo primarily consist of ornamental items, such as several necklace beads, golden earrings, and an ivory comb, all made from materials of different natures (stone, metal, shell, and ivory) (Fig 15) (Table 4).

**Table 4:** Graves goods analysed and analytical techniques employed.

Sample Nº	ID	TOMB	DESCRIPTION	MINERALOGICAL COMPOSITION	ANALYSES PERFORMED
001	CT21-C1-UE110–4	I	Cylindrical, planar, whitish-yellow necklace bead with irregular edge	Marine bivalve shell: calcite and aragonite	SM, ESEM-EDX, XRF, Raman
002	CT21-EST 1–4	I	Dark green barrel-shaped necklace bead, highly polished, biconical central perforation	Green mica, with presence of Cr and Fe	SM, ESEM-EDX, XRD, XRF, Raman
003	CT21-C1-EST1/UE110–2	I	Dark green barrel-shaped necklace bead, highly polished, biconical central perforation	Green mica, with presence of Cr and Fe	SM, ESEM-EDX, XRD, XRF, Raman
004	CT21-EST1-UE110–6	I	Spherical silver bead	Silver with surface alteration to chlorargyrite (and its bromine-rich variety: Ag (Cl, Br)	SM, ESEM-EDX, XRD, XRF
005	CT21-EST1-UE110–6	I	Cylindrical silver bead	Silver with surface alteration to chlorargyrite (and its bromine-rich variety: Ag (Cl, Br)	SM, ESEM-EDX, XRD, XRF, Raman
006	CT21-EST1-UE110–6	I	Gold earring	Gold with small amount of silver	SM, ESEM-EDX, XRD, XRF
007	CT21-C1-EST1/UE110–2	I	Ivory comb	Fluorapatite	SM, ESEM-EDX, XRD, XRF, FTIR
008	CT-22-EST2/UE204-(1)	II	Metallic arrow	Copper	XRF

**Fig 15:**
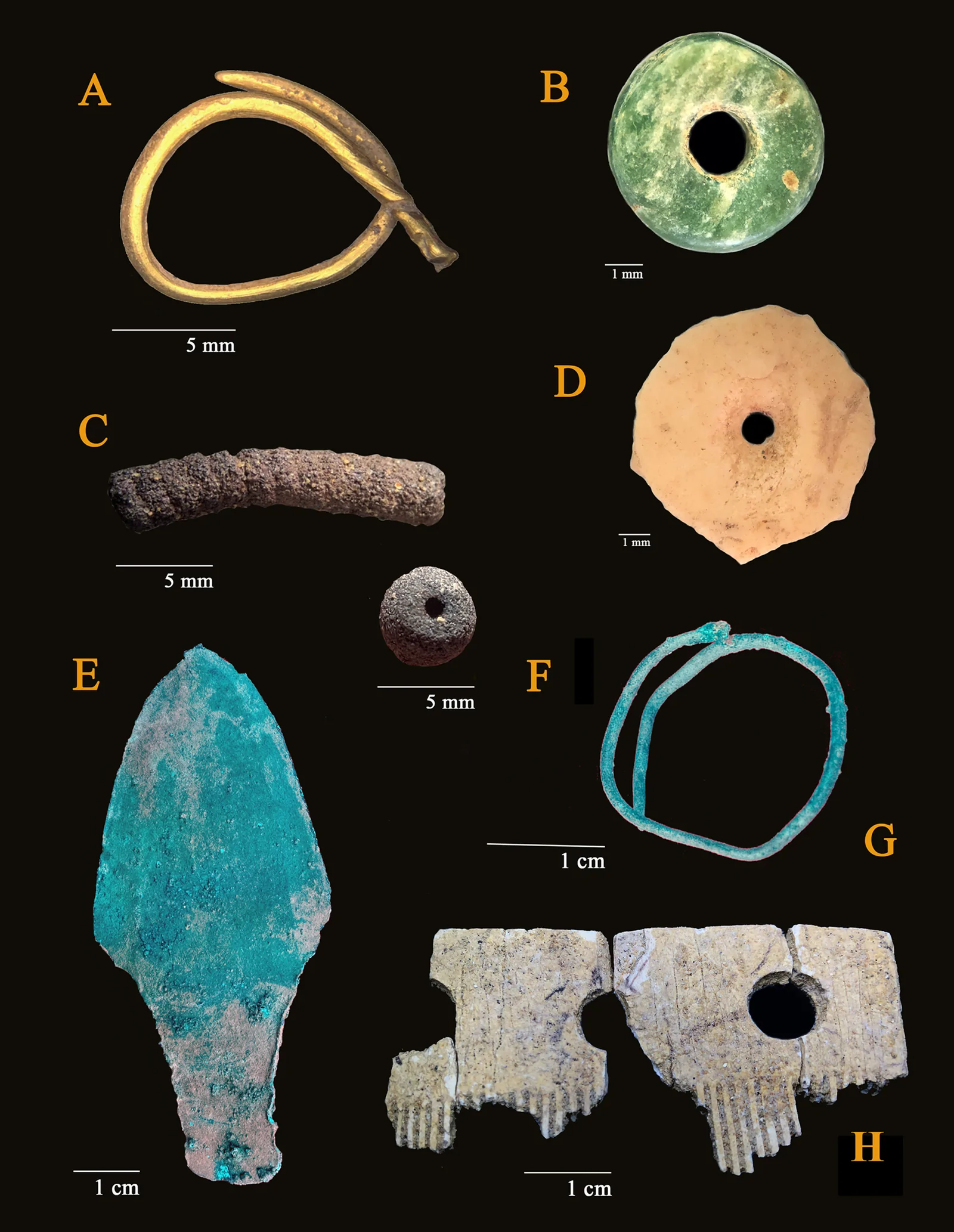
Graves goods Tomb I and II. A. Gold earring; B. Dark green barrel-shaped necklace bead, highly polished, biconical central perforation; C. Spherical silver bead; D. Cylindrical, planar, whitish-yellow necklace bead with irregular edge; E. Metallic arrow; F. Cylindrical silver bead; G. Copper earring; H. Ivory comb.

Among the necklace beads, the whitish-yellow specimen made from a fragment of mollusc shell exhibits a mineralogy characterised by calcite/aragonite, with the typical structure of fine layers of calcium carbonate crystals (Fig 16A,16B). Its chemical composition, in addition to the inherent elements of this material such as calcium, carbon, oxygen and phosphorus, also revealed the presence of some terrigenous impurities, such as silicon, iron, potassium, titanium and zirconium, from the sediment, as well as minerals formed through chemical precipitation (mainly chlorides and sulphates) due to the coastal environment in which it was found (Table 5).

**Table 5:** Chemical composition (in %) of the major and minor elements of the grave goods. For abbreviations, see Table 3.

Sample Nº	001	002	003	004	005	006	007	008
**Artifact**	Marine shell	Rock bead	Rock bead	Silver bead	Silver bead	Gold earring	Ivory comb	Metallic point
Chemical composition								
Ag	–	–	–	65.8732	68.9916	12.2886	–	–
Al_2_O_3_	1.9691	36.6439	36.1207	9.4899	6.5413	–	–	0.3559
As_2_O_3_	–	–	–	–	–	–	–	11.6910
Au	–	–	–	–	–	87.6802	–	0.0014
BaO	–	0.1265	0.1462	–	–	–	–	–
Bi_2_O_3_	–	–	–	–	–	–	–	0.1030
Br	–	–	–	6.3776	5.5304	–	0.0337	–
CaO	90.9726	0.3354	0.2459	9.2969	2.3475	–	62.8260	–
Cl	–	–	–	6.2150	8.6196	–	–	–
Cr_2_O_3_	–	0.2010	0.1950	–	–	–	–	0.0756
CuO	–	–	–	–	0.0330	0.0312	–	83.3136
Fe_2_O_3_	0.4412	2.2424	3.0895	0.1562	1.8659	–	–	0.0452
K_2_O	0.1974	11.8107	12.9710	–	–	–	–	–
MgO	0.7002	–	–	–	–	–	0.1938	–
MnO	0.0571	0.0000	–	–	–	–	–	–
Na_2_O	–	1.6973	1.2579	–	–	–	36.5830	3.5694
P_2_O_5_	1.4292	–	–	–	–	–	–	0.0210
Rb	–	0.0333	0.0316	–	–	–	–	–
Sb_2_O_3_	–	–	–	–	–	–	–	0.4731
SiO_2_	3.7191	46.6328	45.5882	2.5094	5.8461	–	–	0.0507
SO_3_	0.4117	0.0000	0.0120	0.0424	–	–	0.2356	0.1379
SrO	0.0203	0.0387	0.0501	–	–	–	0.1279	–
TiO_2_	0.0690	0.2272	0.2824	–	0.1985	–	–	–
ZnO	–	–	–	–	–	–	–	0.1637
ZrO_2_	0.0131	0.0108	0.0096	0.0394	0.0261	–	–	–

**Fig 16:**
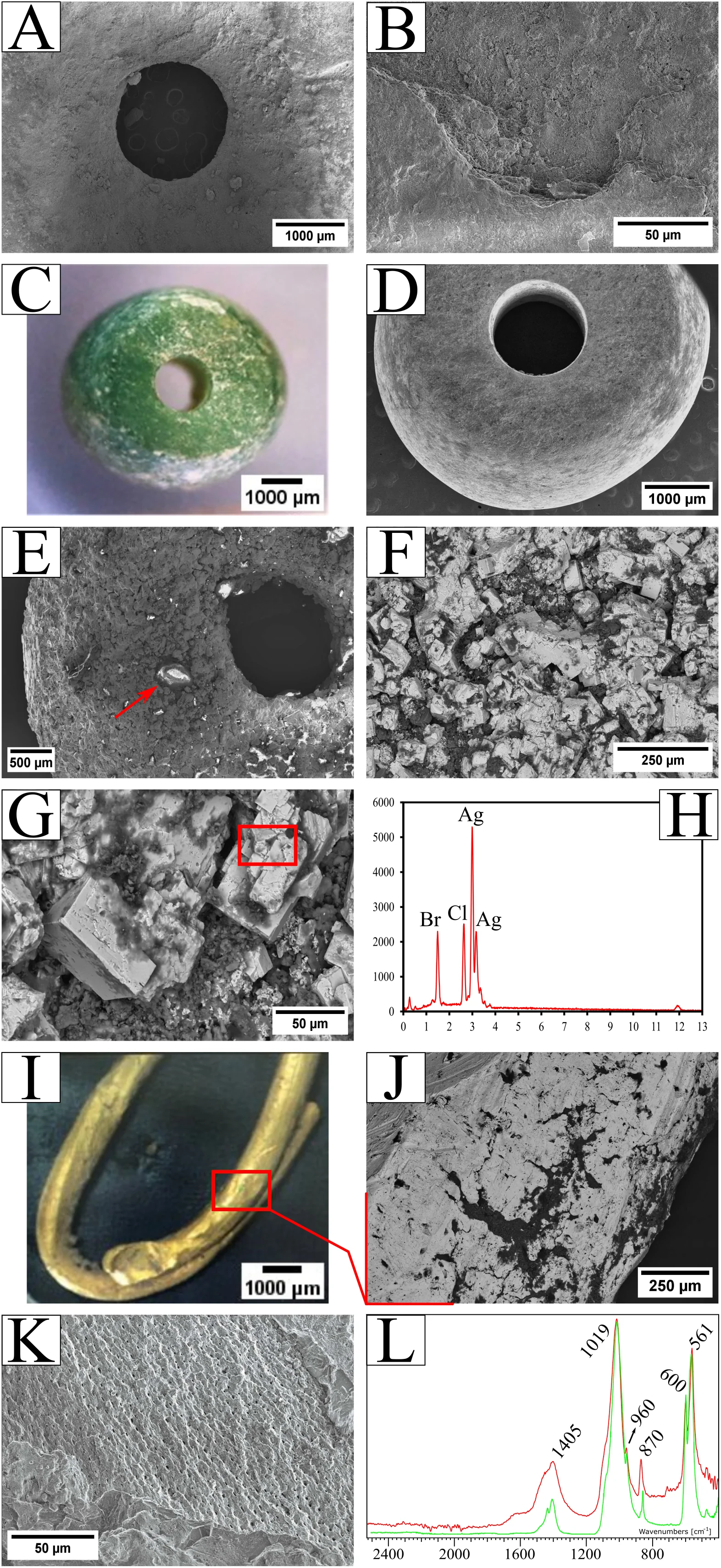
General view (A) and detail of the fine-layered structure of calcium carbonate crystals (B) in the shell bead. Macroscopic general appearance under binocular magnification (C) and detail of the surface with microporosity (D) of the green bead. General view of the surface of the spherical silver bead (E) and detail of the chlorargyrite crystals (F and G) along with corresponding microanalysis (H). General view of the gold earring (I) and detail of the surface showing remains of detrital material (J). Surface detail with micropores on the ivory comb (K) and FTIR spectrum (L) of the sample and the fluorapatite standard (red and green spectra, respectively). The fluorapatite standard (record R050529-1) was obtained from the RRuFF database.

On the other hand, the green beads are composed of silicon, aluminium, potassium and iron, compatible with a silicate from the mica group (confirmed through XRD and Raman), with a minor proportion of Ca, Ti, Ba, Sr and Rb attributed to sediment grains adhered to the surface. Chromium was also detected, together with Fe, could act as chromophoric elements responsible for the characteristic green colour of these beads (Table 5). Additionally, sodium, chlorine and sulphur were identified, likely corresponding to saline impurities from the marine environment. The green colour and the presence of chromium along with Fe enable this material (Si, Al, K, Fe) to be defined as a muscovite. These green beads, although displaying some tonal variation (Fig 16C), are generally quite homogeneous from a mineralogical point of view, with small pores observed on their surfaces, likely caused by dissolution processes (Fig 16D).

Regarding the third group of necklace beads, the metallic ones, these are characterised by a silver composition (Table 5). However, due to taphonomic processes during burial, the silver has altered into secondary phases such as chlorargyrite, readily observable by SEM, particularly because its cubic habit (Fig 16E, 16F, and 16G) and confirmed through XRD, with variable amounts of bromine, which may be substituting for Cl, or may be present as a bromine-rich variety, bromian chlorargyrite (Fig 16H). Terrigenous impurities are also observed (red arrow, Fig 16E), specifically quartz grains (beach sand). This composition is consistent with the alteration in a coastal–marine environment of necklace beads originally made of silver.

The gold earring (Fig 16I) is composed entirely of gold, with traces of silver (Table 4), and no other impurities were detected in the alloy, apart from adhered sediment deposits on its surface (Fig 16J, dark material). The gold (87.68%) and silver (12.3%) concentrations are consistent with gold objects from the Southwest, such as Cabezo Juré and Valencina de la Concepción [40,41], as well as from the Northwest of the Iberian Peninsula [42]. This similarity suggests a standardized approach to alloy preparation across these different sites.

The ivory comb shows a porous structure characteristic of this material (Fig 16K), with a composition based on phosphorus and calcium (Table 4), consistent with calcium phosphates of the apatite group. The FTIR spectrum matches with hydroxyapatite (Fig 16L), exhibiting peaks at 1405, 1019, 870, and 600 cm^−1^ which are respectively attributed to asymmetrical stretching of CO_3_^2–^ (carbonate), asymmetrical stretching of PO_4_^3-^ (phosphate), bending of CO_3_^2–^ (carbonate), and asymmetrical bending of PO_4_^3-^ (phosphate). The peaks related to organic compounds typically occur in the 1250–1550 cm^-1^ range, however only those corresponding to the inorganic fraction were detected due to burial processes. In this sample, elements such as magnesium, strontium, sulphur, bromine, and chlorine were also detected (terrigenous impurities, carbonates, marine sulphates and chlorides) (Table 5).

Finally, a small fragment of a metallic arrowhead from the tumulus of Tomb II was analysed by XRF (Table 5). The results show the classic pattern of these pieces made from copper, highlighting, in addition to its presence, arsenic at around 11%, being an arsenical copper. Bismuth and tin were also detected in low proportions, both possibly linked to arsenic. On the other hand, the rest of the elements can be associated either with the composition of the adhered sediments or salts, for example, sodium from halite. Although no chlorine was recorded in the internal part of the fragment, where the cut was fresh, it was detected in the external surface where the alteration is prominent.

After considering the geological, petrological and mineralogical context of the Bay and Coastal region of Cádiz, and comparing this information with the mineralogical and chemical characterisation data of the grave goods analysed, it can be stated that most of the objects are imported materials, clearly exotic to this area. In the case of the green necklace beads, their provenance remains unknown, but their micaceous composition and the presence of rare elements such as chromium suggest an origin associated with igneous or metamorphic rocks, which are absent in the local geology. Therefore, their origin must lie in geologically distant environments. For the silver necklace beads, connections can be proposed with the eastern part of the Iberian peninsula (Argar culture) or with the Iberian Pyrite Belt (SW Iberian peninsula), although isotopic analyses would be required to confirm this (the small size of the gold and silver items has prevented isotopic analyses from being undertaken due to their destructive nature). Regarding the origin of the gold, it is more plausible that it comes from within the Iberian peninsula, possibly linked to placer-type deposits of the Iberian Massif, although an African origin should not be ruled out, particularly if its appearance can be associated with the items made of ivory. As for the ivory comb, an African provenance is likely, although a possible Asian origin cannot be excluded.

### 4.5 Ceramic studies

Pottery is the most common archaeological material found in the necropolis. Most specimens were collected from the superficial layers of the burial structures. It is highly fragmented and no whole pieces have been found. The total count (NFR) is 687 ceramic fragments, of which 141 correspond to rims (MNI); 434 fragments were found in Tomb I, with 92 rims (21.2%), across nine contexts, which mostly correspond to the tumulus stratigraphic layers (UEs 100–107). The fill of the hypogenic chamber (UE 110) yielded 108 small fragments. Tomb II yielded 253 fragments, with 49 rims (19.36%), and an aggregate weight of 2,647 g (10.46 g on average per fragment) (Table 6).

**Table 6:** Count of ceramic fragments per stratigraphic unit, identified MNI, and total weight of fragments.

TOMB I				TOMB II			
UE	NFR	MNI	Weigth (gr.)	UE	NFR	MNI	Weight (gr.)
100	118	28	1847	202	15	3	284
102	42	4	378	203	128	26	1474
103	16	2	482	204	110	20	889
104	16	5	265	**TOTAL**	**253**	**49**	**2647**
105	10	3	159				
107	16	2	210				
108	76	20	983				
109	32	6	301				
110	108	22	1089				
**TOTAL**	**434**	**92**	**5714**				

For the typological analysis, rim fragments were selected (N = 141), of which 85 (60.3%) presented features on which formal attributions could be based. The remaining 56 (39.7%) were too small for typological characterisation. A typology was developed based on formal analysis (Fig 16); each form is based on basic features, such as having a simple (without any inflection) or more complex profile (for instance, having a carinated wall or a narrowing body line). The forms are named based on their traditionally attributed function. We are aware that typologies based on formal characteristics are rather subjective, but the state of preservation of the assemblage does not allow for a comprehensive morphometric study.

The three main identified forms are subdivided into types, based on the wall’s profile (open-closed), the relationship between estimated height and diameter of the mouth, and height of the inflection point (Fig 17).

**Fig 17:**
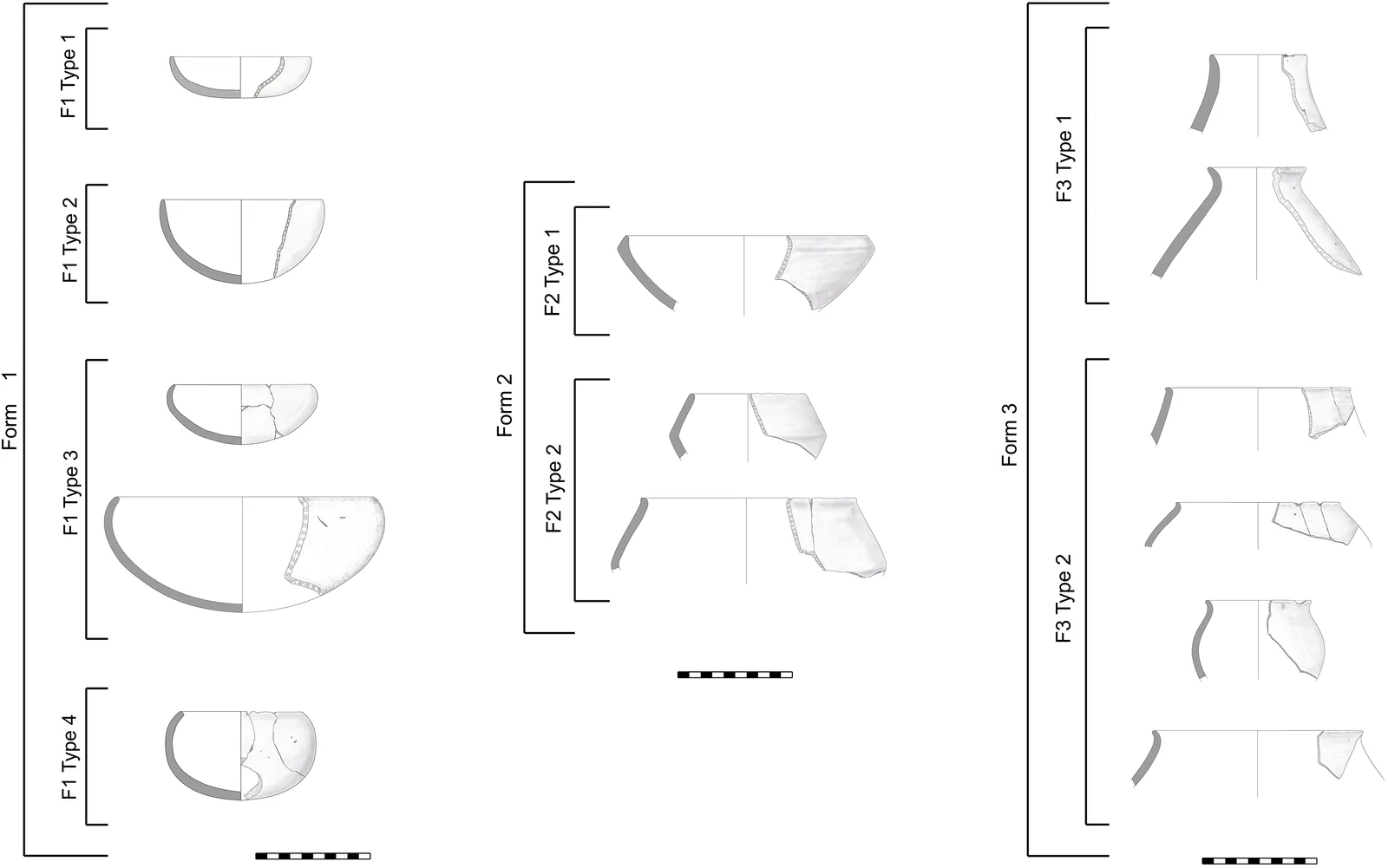
Typological diagram of the ceramics from the Trafalgar necropolis.

Form I, bowls with a simple profile and a convex base, is the most widely represented with 54 specimens (63.5%); spherical Type 1.1 accounts for fourteen examples (26%); open and hemispherical Type 1.2 presents 22 specimens (40.7%); hemispherical close Type 1.3 accounts for eight examples (14.8%); and spherical/globular Type 1.4 bowls is found ten times (18.5%). Carinated vases (Form 2) are represented by twelve specimens (14.1%). Form 3 corresponds to globular vessels which are taller than the mouth is wide, with an ovoid body and a convex base, and accounts for nineteen examples (22.4%). For the case of bottles, deep containers with straight or slightly curved walls and a narrow neck that is distinct from the body (Type 3.1) appear three times (15.8%). Globular vessels with an outfolding rim (Type 3.2) account for 84.2% of the group, with sixteen examples (Table 7).

**Table 7:** Percentage distribution of forms and types.

		Tomb I		Tomb II	
FORM	TYPE	MNI	%	MNI	%
1	1.1	12	21,8	2	6,7
	1.2	12	21,8	10	33,3
	1.3	7	12,73	1	3,3
	1.4	7	12,73	3	10
2	2.1	1	1,8	4	13,3
	2.2	4	7,27	3	10
3	3.1	2	3,6	1	3,3
	3.2	10	18,2	6	20

In the assemblage, closed shapes are slightly more numerous than open ones, particularly forms related to food consumption and presentation. Although larger pans are also represented, small or medium-sized specimens predominate (although some may exceed 20 cm in diameter). Typologically, the assemblage fully matches other Early Bronze Age assemblages from the region [36,43,44].

From a technological perspective, it must be noted that our microscopic analysis is based only on fractures and surfaces. The lack of whole specimens, on the other hand, stands as a significant limitation. Fabrics have a predominantly sandy matrix, but with abundant medium-grain inclusions. These vary; while in some samples they present greater sphericity (Fig 18a and 18c) in others they are more angular (Fig 18a and 18d). Macroscopic analysis with a magnifying glass reveals the presence of seashell inclusions (Fig 18a). The wares mostly present indications of mixed firing, with reddish and dark tones. The surfaces were polished, with few burnished examples, and even most of these only have this treatment in the interior. No decorated wares were found.

**Fig 18:**
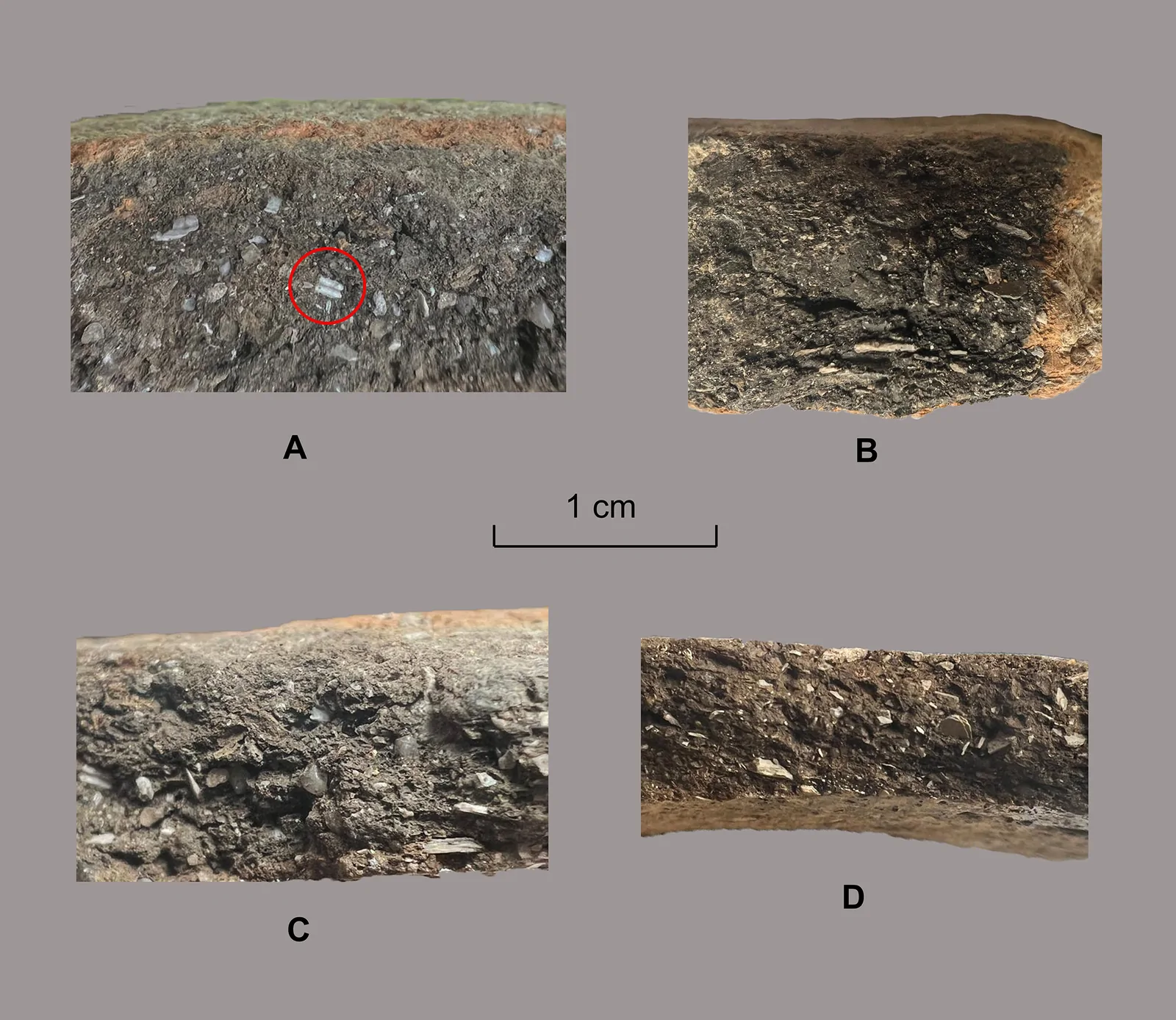
Examples of ceramic fabrics from Trafalgar. The red circle marks the fragment of seashell used as tempering.

## 5. Discussion: the late builders of megaliths in the south of the Iberian peninsula?

The construction of the last megalithic monuments in the south of the Iberian peninsula is hard to pinpoint with precision. Despite the numerous constructions dated to this final stage, radiocarbon dating has not been applied systematically in the region. The process is framed by the end of Chalcolithic societies documented in the south-west of the Iberian peninsula from around 2300 BC [45–47], when major settlements between the Algarve and the Strait of Gibraltar are abandoned or radically change. In Cadiz, specifically, this phenomenon led to the emergence of smaller settlements, which suggests a profound reconfiguration of the social system. In this sense, Chalcolithic structures, based on a markedly communal ideology, yielded to social formations characterized by increasing inequality and stratification. Some authors have linked this generalised crisis in 2400–2200 BC with the 4.2 ka BP event, a climate change episode that, in the south of the Iberian peninsula, led to a greater aridity and a significant transformation of the landscape [48,49].

During the initial stages of the Bronze Age, the consolidation of progressively more hierarchical societies and the articulation of pre-state or state political models can be verified, as illustrated in the south-east of the Iberian peninsula by the culture of El Argar [5] and also in the south-west [50]. These sociopolitical transformations impacted the funerary context, where collective practices were gradually replaced by individual cist burials. Between ca. 2200 and 2100 cal BC, a meaningful reduction in the monumental scale of megalithic constructions is accompanied by a marked decline in their frequency; this phenomenon is simultaneous to the emergence of necropolises of individual burials, represented by graves, cists, and hypogea, among others.

The necropolis of Trafalgar (Barbate, Cadiz) stands as a key reference for understanding the final stage of the megalithic phenomenon in southern Iberia. The radiocarbon evidence points to a relatively short but intense period of funerary activity, broadly between ca. 1875 and 1600 cal BC, followed by an isolated later deposition or reuse episode in the corridor of Tomb I dated to 1610–1440 cal BC. The Trafalgar sequence indicates the use of megalithic tombs during the Bronze Age, with funerary activity concentrated within only a few generations. In this framework, Tomb I is coeval to other mixed hypogea in the area of Cadiz, such as tomb 1–2 in Los Algarbes [51], although the initial date in Trafalgar is more recent. Likewise, its development recalls that of the Alcaide necropolis (Antequera), although Trafalgar seems to have been abandoned earlier, because in Alcaide some hypogea remained in use until well into the 2^nd^ millennium cal BC (Table 7) [52].

In addition, the *tholos* of Trafalgar is one of the latest of its type [53], with a chronological range between 1875 and 1630 BC. This date differs markedly from those of other *tholoi* in the region, which were built in the 3^rd^ millennium BC, for instance in Peñarroyo I, active around 2100 BC [54], Centirã [55], Huerta Montero [56], and Alcalar [57]. In the wider context, the period 2200−2100 BC witnesses a diversification of funerary manifestations in the south of the Iberian Peninsula [2,6,7]. In this period, a major drop in the use of dolmens runs parallel with a reduction in their monumental scale [58] and the reuse of older tombs, as significantly illustrated by Tesorillo de la Llaná [59], Los Gabrieles [60], and Viera [61], among others. At the same time, the construction of megalithic-inspired cists is attested in such necropolises as La Traviesa (2020−1400 cal BC) [62], El Becerrero, and El Castañuelo [63] in Huelva, and Llano de la Virgen [64] in Málaga (Table 8). In addition, the use of peripheral areas in extant monuments has been recurrently attested [2].

**Table 8:** Megalithic funerary structures of the late 3^rd^ millennium BC and the early 2^nd^ millennium BC with radiocarbon dates in Cádiz and Málaga.

Archaeological site	Location	Type	Lab. code	Date (BP)	Material	Reference
Centirã	Serpa (Beja)	Tholos	Sac-2796	3710 ± 45	Human bone	[55]
Centirã	Serpa (Beja)	Tholos	Sac-2789	2950 ± 80	Human bone	[55]
Centirã	Serpa (Beja)	Tholos	Beta-331980	3680 ± 30	Human bone	[55]
Centirã	Serpa (Beja)	Tholos	Sac-2782	3760 ± 70	Human bone	[55]
Centirã	Serpa (Beja)	Tholos	Sac-2792	3790 ± 110	Human bone	[55]
Centirã	Serpa (Beja)	Tholos	Sac-2788	3810 ± 80	Human bone	[55]
Centirã	Serpa (Beja)	Tholos	Sac-2790	3900 ± 45	Human bone	[55]
Centirã	Serpa (Beja)	Tholos	Sac-2791	3940 ± 50	Human bone	[55]
Alcalar 7	Portimão (Faro)	Tholos	Beta-180982	3280 ± 40	Charcoal	[57]
Alcalar 9	Portimão (Faro)	Tholos	Beta-316624	3730 ± 30	Charcoal	[57]
Huerta Montero	Almendralejo (Badajoz)	Tholos	GrN-16954	3720 ± 100	Human bone	[56]
Pozuelo 6	Zalamea la Real (Huelva)	Dolmen	3580 ± 120	3580 ± 120	Charcoal	[77]
Pozuelo 4	Zalamea la Real (Huelva)	Dolmen	CNA-2581.1.2	3760 ± 35	Charcoal	[78]
Pozuelo 1	Zalamea la Real (Huelva)	Dolmen	CNA-3266.1.1	3723 ± 34	Charcoal	[78]
Pozuelo 1	Zalamea la Real (Huelva)	Dolmen	CNA-3260.1.1	3706 ± 34	Charcoal	[78]
Pozuelo 3–4	Zalamea la Real (Huelva)	Dolmen	CNA-2598.1.1	3437 ± 30	Charcoal	[78]
Pozuelo 1	Zalamea la Real (Huelva)	Dolmen	CNA-3265.1.1	3303 ± 35	Charcoal	[78]
Pozuelo 3	Zalamea la Real (Huelva)	Dolmen	CNA-2587.1.1	3770 ± 35	Charcoal	[78]
Pozuelo 3	Zalamea la Real (Huelva)	Dolmen	CNA-2585.1.1	3660 ± 35	Charcoal	[78]
Pozuelo 3	Zalamea la Real (Huelva)	Dolmen	CNA-2586.1.1	3640 ± 35	Charcoal	[78]
Pozuelo 3	Zalamea la Real (Huelva)	Dolmen	CAMS-2588.1.1	3101 ± 30	Charcoal	[78]
Los Gabrieles 4	Valverde del Camino (Huelva)	Dolmen	Beta-185650	3700 ± 50	Charcoal	[60]
La Orden-El Seminario 7016	Huelva	Hipogeum	CNA-622	3600 ± 60	Human bone	[6]
La Orden-El Seminario 7016	Huelva	Hipogeum	CNA-327	3795 ± 50	Human bone	[6]
La Orden-El Seminario 7055	Huelva	Tholos	CNA-330	3700 ± 50	Human bone	[6]
Puerto de los Huertos	Berrocal (Huelva)	Dolmen	CNA-341	3680 ± 50	Charcoal	[60]
Los Algarbes 1-2	Tarifa (Cádiz)	Hipogeum	Beta-564943	3300 ± 30	Human bone	[51]
Peñarroyo I	Benalup- Casas Viejas (Cádiz)	Tholos	Beta-630563	3700 ± 30	Human bone	[54]
Peñarroyo I	Benalup- Casas Viejas (Cádiz)	Tholos	Beta-632843	3570 ± 30	Human bone	[54]
Piedras Blancas	Antequera (Málaga)	Dolmen	Beta-613205	3770 ± 30	Human bone	[79]
Piedras Blancas	Antequera (Málaga)	Dolmen	Beta-591811	3520 ± 30	Human bone	[79]
Viera	Antequera (Málaga)	Dolmen	Beta-353822	3580 ± 30	Human bone	[63]
Cerro de la Corona	Totalán (Málaga)	Dolmen	Beta-93020	3490 ± 120	Bone	[80]
Tesorillo de la Llaná	Alozaina (Málaga)	Dolmen	GrN-26488	3250 ± 40	Human bone	[59]
Tesorillo de la Llaná	Alozaina (Málaga)	Dolmen	GrN-26475	3250 ± 50	Human bone	[59]
El Tardón A	Antequera (Málaga)	Dolmen	UGRA-260	3530 ± 60	Human bone	[81]
El Tardón B	Antequera (Málaga)	Dolmen	GrN-16066	3745 ± 25	Human bone	[81]
Llano de la Virgen	Coín (Málaga)	Cist	GrN-19990	3660 ± 40	Human bone	[64]
Alcaide 14	Antequera (Málaga)	Hipogeum	CNA-2275	3475 ± 40	Human bone	[82]
Alcaide 14	Antequera (Málaga)	Hipogeum	GrN-16061	3180 ± 100	Human bone	[82]
Alcaide 14	Antequera (Málaga)	Hipogeum	CNA-2274	3115 ± 40	Human bone	[82]
Alcaide 15	Antequera (Málaga)	Hipogeum	GrN-16064	3340 ± 90	Human bone	[82]
Alcaide 15	Antequera (Málaga)	Hipogeum	SUERC-69030	3055 ± 25	Human bone	[47]
Alcaide 15	Antequera (Málaga)	Hipogeum	SUERC-72359	3041 ± 32	Human bone	[52]
Alcaide 9	Antequera (Málaga)	Hipogeum	SUERC-72355	3123 ± 32	Human bone	[52]
Alcaide 1	Antequera (Málaga)	Hipogeum	Beta-625819	3370 ± 30	Human bone	[52]
Alcaide 1	Antequera (Málaga)	Hipogeum	Beta-625818	3480 ± 30	Human bone	[52]

The identification of megalithic constructions built *ex novo* during the 2^nd^ millennium BC poses a significant challenge, owing to the lack of absolute dates and the difficulty of establishing the moment when the structures were first built [2]. In this regard, we propose as a hypothesis that the Trafalgar tombs were constructed during the Bronze Age, but this hypothesis can only be confirmed through OSL dating. The sixteen radiocarbon dates on human bone suggest that both graves of the necropolis were active simultaneously between 1875 and 1625 cal BC. Although both tombs were active at the same time, the use of Tomb II appears to have been concentrated within only a few decades according to the statistical model. This may be related to the structural instability of the tomb’s roof, as suggested by the documented architectural modification. Such instability may have contributed to the cessation of funerary depositions inside the chamber, whereas in Tomb I the inhumations and the rearrangement of skeletal assemblages continued over a longer period of time. Despite variations in the architectural design and internal arrangement, both were coeval, which, together with the presence of primary and secondary burials and common post-depositional practices (e.g., manipulation of remains), indicates a culturally homogeneous community that managed a funerary space that was closely integrated with the landscape. This is supported by the material record, which is consistent with the ^14^C radiocarbon dates and the absence of elements from the 2^nd^ millennium BC, as confirmed by the survey undertaken in the promontory [13].

Finally, other examples of this continuity in the south-west include the dolmen of El Carnerín (Alcalá del Valle), which has been described as a one-phase site dated to the 2^nd^ millennium BC, owing to the lack of absolute dates [65], and Loma del Puerco in Chiclana [66,67]. In the Malaga area, the necropolis of El Tardón has also been put forward as an example of the persistence of megalithic construction, although the dates from Tomb A (2117−1642 cal BC) could be the product of reuse during the Bronze Age, as suggested by its architectural and materials similarities with Tomb B, dating to the bell-beaker period [68]. Finally, in the south-east of the Iberian Peninsula, the necropolis of Los Eriales (Granada) stands out [69]; there, recent Bayesian analyses on 30 dates span the late 3^rd^ millennium cal BC and the 18^th^ century BC. It has been interpreted as sign of resistance of non-Argaric communities against increasing social stratification in the Argaric world [70].

However, even if a Bronze Age chronology is proposed for the construction of the Trafalgar necropolis, the alternative hypothesis that the chambers were cleared of skeletal remains from an earlier phase should not be completely ruled out. Similar processes have been suggested for Cerro Malhanito and Roça do Casal do Meio, where the removal of the earlier primary burials appears to have erased evidence of their original use, leaving traces only of later reuse [71,72].

In Cadiz, there are barely any documented individual burials for the 2^nd^ millennium BC, except for three single grave burials in El Berrueco (Medina Sidonia) [73] and a single grave burial in El Estanquillo [74], all of which lack radiocarbon dates. Unlike the south-east and south-west, where individual burials in cists emerge in force, Cadiz is characterized by the persistence of collective burials during the 2^nd^ millennium BC: Loma del Puerco (Chiclana de la Frontera) [66,67], Los Algarbes (Tarifa) [51,75], and Bray’s Cave (Gibraltar) [76]. They are all clear examples of this continuity, although using different funerary typologies (natural caves, artificial caves, and fossa). We hypothesize that this is a phenomenon of resistance against new centralised political systems, as the region lacks other indications of pre-state organizational structures.

Beyond the architectural features, the links with megalithic constructive traditions are also proven by the undeniable relationship between the necropolis and the surrounded landscape. The necropolis of Trafalgar is conceived as a visual and symbolic dialogue between land and sea, where megalithic structures are assimilated to a liminal space of great natural “power”, which the communities that built these tombs appropriated [83–85]. In terms of visual perception and the relationship with the natural landscape, far from dwelling areas, this stands out from the cultural phenomenon reflected by Bronze Age cists, which are often located within the immediate surroundings of the villages.

The grave goods, although scarce, stand out for their craftsmanship and distinctive exotic character. The comparison of the region’s geological, petrological, and mineralogical resources and the pieces’ chemical characterization leads to the conclusion that a substantial proportion of these were not local. The green necklace beads are illustrative of this: their mica composition and presence of chromium suggests an igneous or metamorphic source, which are absent from the local geology, indicating that they were brought from far away.

Similarly, the silver beads present compositional features that point to the east of the Iberian peninsula, in the regions under the influence of the El Argar culture, or to the Iberian Pyrite Belt. A more precise provenance would require isotopic analyses.

The circulation of goods suggested by these finds is framed within a context of far-reaching social change in the transition between the Chalcolithic and the Bronze Age. As suggested by Montero and Murillo-Barroso [86], the social value of metals was not fully consolidated until the early Bronze Age, when individuals began to overtake community ideologies. In this setting, metals, and specifically ornaments, became a key instrument to materialise inequality and build social hierarchy [87]. While in El Argar there was mass silver consumption, probably leading to an early system of weights and measures under state control through the use of standardised objects [88,89], in Cadiz and Malaga the use of this metal was very limited. In the southernmost region of the Iberian peninsula, silver objects appear mainly as ornaments (bi-conical beads, rings, and spirals), but are scarcely present in tombs (Table 9). This disparity suggests that, in this region of the Iberian peninsula, elite individualisation strategies were less intense, encountering stiffer opposition to changes in kinship-based social structures [53].

**Table 9:** Presence of gold and silver objects in Bronze Age collective burials of the Bronze Age in the southernmost region of the Iberian Peninsula (provinces of Málaga and Cádiz).

Tomb	Town/ Province	Type	gold and silver artifacts	Reference
Alcaide 9	Antequera (Malaga)	Hipogeum	Silver: 1 Diadem 3 spirals	[52]
Tesorillo de la Llaná	Alozaina (Malaga)	Dolmen	Silver: 5 spirals 3 simple rings	[59]
Cerrete de la Cañada de Algane	Coín (Malaga)	Dolmen	Silver: 1 spiral	[59]
Corominas 4	Estepona (Malaga)	Dolmen	Gold: 1 espiral	[81]
El Carnerín	Alcalá del Valle (Cadiz)	Megalithic cist	Silver: 2 simple rings 1 spiral	[90]
Peñarroyo I	Benalup- Casas Viejas (Cadiz)	Tholos	Gold: 1 sheet Silver: 1 spiral	[54]
Los Algarbes 5	Tarifa (Cadiz)	Hipogeum	Gold: 1 simple ring 1 decorated sheet	[75]
Las Cumbres I	Puerto de Santa María (Cadiz)	Hipogeum	Gold: 2 simple rings Silver: 6 spirals 1 single ring 4 biconical beads 2 copper knives with silver rivets	[91]
Trafalgar I	Barbate (Cadiz)	Mixed hypogeum	Gold: 2 simple rings Silver: 1 spiral 2 beads	This work

Regarding gold, its sources seem to be mostly connected with alluvial deposits in the Iberian Massif. The analyses of the samples show a 12.3% silver content and residual copper levels of just 0.031%, a chemical signature that suggests the use of unrefined native gold and rules out intentional alloys [41]. This composition finds close parallels in the gold-rich districts of northwestern Iberian peninsula [42], indicating a manufacturing tradition based on the mechanical shaping of natural nuggets. Owing to its brilliance and immutability, gold acted as vehicle of memory and symbol of eternal power [92], although its Chalcolithic sacred-symbolic meaning was to be displaced by expressions of individual prestige in the Bronze Age [41].

Within these circuits of exchange, ivory stood as a high-social-value material. However, the late Chalcolithic witnessed the gradual substitution of metal for ivory, which must not only be understood as indicating a disruption of certain trade routes, but as a shift in the preferences of the elite [93,94]. Finally, it must be noted that the circulation of materials and knowledge was facilitated by the intense cultural connectivity that characterises the Strait of Gibraltar. Between 2300 and 1500 BC, the region was a contact hub that linked the communities of North Africa and the south of the Iberian peninsula within a framework of shared ritual models and ideologies [95–97].

Regarding the ceramic assemblage associated with the Trafalgar burials, it has been suggested that the depositional pattern combines characteristics typical of both accidental deposits and deliberate depositional practices [98]. The assemblage exhibits a high degree of fragmentation, together with the low weight and small size of the sherds recovered from within the graves, the absence of complete vessels, and a large number of fragments that cannot be refitted. Conversely, the spatial proximity of fragments belonging to the same object, the relative homogeneity in post-depositional alterations and fragment size across much of the assemblage, the predominance of fragments within the units composing the mound, and the absence of vessel types associated with storage or bearing traces of thermal alteration linked to food preparation suggest that the assemblage resulted from a conscious social practice.

Although it cannot be determined whether the processes of breakage and deposition occurred during a single event or throughout the period of use of the necropolis, the typological coherence of the assemblage with Early Bronze Age forms indicates no evidence for multiple chronological phases. As noted by A. Blanco-González, the combination of breakage and dispersal processes is common in tumular contexts [98]. The deliberate fragmentation of objects with social functions has been documented elsewhere in Europe during Late Prehistory [99]. The intentional breaking of vessels following their use in funerary rites may have contributed to reinforcing material bonds between the living participants in the ceremonies and the deceased members of the community [100]. It should not be ruled out that the ceramic assemblage was associated with social practices of commensality and ritual fragmentation linked to funerary rites, in which the missing fragments from partially reconstructable vessels may have been retained by the living as “souvenirs” [99].

## 6. Conclusions

Trafalgar represents the continuation of monumental megalithism during the first half of the 2^nd^ millennium BC. Tomb I, which has a mixed structure, comprises an orthostatic corridor that leads to an artificial cave acting a burial chamber. Its closest parallel can be found in Tomb 1–2 in the necropolis of Los Algarbes (Tarifa, Cadiz), integrated by an artificial cave and a corridor, partly cut into the rock and partly built with orthostates and covering slabs [101]. Tomb II is a *tholos* singled out by the three-orthostate pillar that supports the main chamber, suggesting the collapse and repair of the structure, which is unsurprising, since the structures are built on fairly unstable dune terrain. Parallels for *tholoi* featuring structural stone or wooden pillars to support the chamber roof, or reinforcement walls, are found throughout the southern Iberian peninsula, from Almería to Portugal. At Los Millares [102] and El Barranquete [103], pillars and walls were documented reinforcing the stability of the corbelled vault in several of the burials. The earliest radiocarbon dates (1875–1700 cal BC) of Tomb II are the most recent for the type in the south of the Iberian peninsula [48]. What distinguishes Tomb II is that its construction was driven by structural instability, whereas in other *tholoi* across the Iberia, such elements were part of the original architectural project.

The burial practices documented in the two Trafalgar megaliths are typical of funerary megalithic expression in the south of the Iberian peninsula. These were collective tombs (thirteen individuals in Tomb I and seven in Tomb II), active simultaneously during a 240-year period.

In general terms, it can be concluded that, in the south-west of the Iberian peninsula, the transition to the early Bronze Age is expressed through the appearance of individual cists, which means a formal departure from earlier collective megalithic traditions. However, this process was not uniform; in different areas [1,2,4,53], like the southernmost region of the Iberian peninsula, the continued construction of megaliths is documented, and the reuse of ancient dolmens coexisted with new burial practices. These collective burial practices suggest ideological and cultural resistance by communities in the Strait of Gibraltar to the social individualisation processes that were under way in other regions. Instead of fully adopting the new individualist paradigm, these groups chose to uphold and adapt Chalcolithic burial traditions, thus reaffirming their traditional social structures [2, 3]. The data obtained at Trafalgar contribute significantly to the broader debate on the diverse trajectories of megalithic monumentality during the Bronze Age. Rather than a homogeneous phenomenon, the regional record displays multifaceted practices, including the reuse of collective tombs as a mechanism of social resistance [53] or legitimation [6], the deliberate destruction of dolmens to condemn memory [60], and the regional recreation of the megalithic tradition through cists, small dolmens [4,104,105], or innovative terraced enclosures [78]. The data from Trafalgar are highly consistent with the hypothesis of a brand-new megalithic space founded in the early 2^nd^ millennium BC, reinforcing its unique character in the prehistoric record of the south of the Iberian peninsula.

Last but not least, the issue of preservation of this form of monumental heritage must also be addressed. The necropolis is located in an exceptional natural environment (La Breña and Marismas del Barbate Natural Park), a dune landscape that, although it has greatly contributed to the outstanding preservation of the site, it now poses major challenges to its valorisation. Owing to the strong winds that batter the Strait of Gibraltar, keeping the features from being covered by the dunes would require constant maintenance. In addition, its location in a very isolated area makes it vulnerable to acts of vandalism. In addition to being a key research tool for constructive analyses, renders have contributed to the generation of 3D models with which to make sketchfab videos (Supplementary Material) and 3D mock-ups with educational purposes.

### Declarations

All necessary permits were obtained for the described study, which complied with all relevant regulations.

## Supporting information

S1 Fig Floor plan and cross-sections of Tomb I. (JPG)

S2 Fig Floor plan and cross-sections of Tomb II. (JPG)

S1 Table Collagen quality. (DOCX)

S1 File Bone sample preparation, collagen extraction, and AMS radiocarbon dating protocol. (DOCX)
