# Supplementary material for: The last builders of megaliths in the South of the Iberian peninsula? The Bronze Age necropolis of Trafalgar (Barbate, Cádiz, Spain)
Source: PLoS One. 2026 Nov 11;21(11):e0359220. doi: 10.1371/journal.pone.0359220 (PMC13754171; doi:10.1371/journal.pone.0359220)
Supplement: Supplementary file 1 [file pone.0359220.s003.docx]

| Reporting No | C/N | %C | %N |
| --- | --- | --- | --- |
| SUERC-110530 | 3,4 | 40.4 | 13.8 |
| SUERC-110531 | 3,4 | 40.8 | 14.2 |
| SUERC-110532 | 3,3 | 38.8 | 13.6 |
| SUERC-110533 | 3,4 | 42.8 | 14.8 |
| SUERC-110534 | 3,3 | 40.7 | 14.6 |
| SUERC-110535 | 3,4 | 39.4 | 13.5 |
| SUERC-110539 | 3,5 | 41.8 | 14.0 |
| SUERC-110540 | 3,4 | 40.9 | 14.2 |

**Tabla S1. Collagen Quality.**
