# Supplementary material for: The last builders of megaliths in the South of the Iberian peninsula? The Bronze Age necropolis of Trafalgar (Barbate, Cádiz, Spain)
Source: PLoS One. 2026 Nov 11;21(11):e0359220. doi: 10.1371/journal.pone.0359220 (PMC13754171; doi:10.1371/journal.pone.0359220)
Supplement: Supplementary file 2 [file pone.0359220.s004.docx]

**SUPPLEMENTARY INFORMATION.**

**THE LAST BUILDERS OF MEGALITHS IN THE SOUTH OF THE IBERIAN PENINSULA: THE BRONZE AGE NECROPOLIS OF TRAFALGAR (BARBATE, CÁDIZ, SPAIN)**

Eduardo Vijande-Vila^1^, Serafín Becerra-Martín^1,2^, Berta Morell-Rovira^3^, Marta Díaz-Zorita Bonilla^4,5^, Eduardo Molina-Piernas^6^, Alejandro Muñoz-Muñoz^1^, Adolfo Moreno-Márquez^7^, Pablo González-Fernández^5^, Salvador Domínguez-Bella^6^, María Leticia Gómez-Sánchez^1^, Diego Salvador Fernández-Sánchez^8^, José Luis Ramírez-Amador^6^, José María Corona-Borrego^9^, María Carmona-Gil^9^, Jesús Corrales Díaz^1^ y Juan Jesús Cantillo-Duarte^1*^

^1^ Departamento de Historia, Geografia y Filosofía, Universidad de Cádiz, Cádiz, Spain.

^2^ Museo de Teba, Málaga, Spain.

^3^ Seminar on Prehistoric Studies and Research (SERP), Universitat de Barcelona, Barcelona, Spain.

^4^ Institut für Ur- und Frühgeschichte und Archäologie des Mittelalters, Tübingen, Deutschland.

^5^ Departamento de Prehistoria y Arqueología, Universidad de Granada, Granada, Spain.

^6^ Departamento de Ciencias de la Tierra, Universidad de Cádiz, Cádiz, Spain.

^7^ Departamento de Geografía, Historia y Humanidades, Universidad de Almería, Almería, Spain.

^8^ Departamento de Prehistoria, Historia Antigua y Arqueología, Universidad Complutense de Madrid, Madrid, Spain.

^9^ Professional archaeologist.

* Corresponding author E-mail: [jesus.cantillo@uca.es](mailto:jesus.cantillo@uca.es)

**1. Bone sample preparation, collagen extraction, and AMS radiocarbon dating protocol.**

Bone samples (1g) were taken from 10 mandibles using a rotary tool with a cutting disc. Bone samples were then sent and analyzed at the Scottish Universities Environmental Research Centre (SUERC) in Glasgow, where they were pretreated for radiocarbon dating, following [1]. Collagen extraction was carried out using a modified version of the [2] protocol. Bone samples were placed into 100 mL of 1M HCl for at least 24 hrs. to dissolve the bone apatite. After the reaction, the excess acid is decanted, and the sample is rinsed in ultrapure water. The remaining sample is then solubilized by being added to 100 mL of ultrapure and heated in a sand bath at 80°C for 3h. The resulting solution is cooled at room temperature and filtered using GF/A filter (Whatman) paper. Finally, the solution is dried down to <20 mL and transferred to a freeze-dryer until the solution is removed and the collagen is left as a white/off-white crystalline powder.

After collagen extraction, samples were dated using accelerator mass spectrometry (AMS). The SUERC operates two AMS instruments: a National Electrostatics Corporation (NEC) 5MV tandem accelerator mass spectrometer and a 250 kV single-stage accelerator mass spectrometer (SSAMS) [1]. Each batch of samples is divided into 13 groups of 10 samples; each group has 3 standards (an oxalic acid II primary standard, a humic acid secondary standard, and either a barley mash or a background secondary standard) [1]. The background of the AMS instrument is monitored routinely by measuring 1.5 mg of high-purity natural graphite powder in the last two positions in every batch [1]. ^14^C results were calculated using the background subtraction method by [3]. Radiocarbon dates were calibrated using the internationally agreed atmospheric curve, IntCal 20 [4], and the OxCal v4.4.2 software.

**References**

1. Dunbar E, Cook GT, Naysmith P, Tripney BG, Xu S. AMS 14C Dating at the Scottish Universities Environmental Research Centre (SUERC) Radiocarbon Dating Laboratory – Corrigendum. Radiocarbon. 2016;58(1):233-233. doi:10.1017/RDC.2016.14.
2. Longin R. New Method of Collagen Extraction for Radiocarbon Dating. Nature. 1971; 230: 241–242. <https://doi.org/10.1038/230241a0>.
3. Brown TA, Southon JR. Corrections for contamination background in AMS 14C measurements. Nuclear Instruments and Methods in Physics Research Section B: Beam Interactions with Materials and Atoms. 1997; 123(1): 208-213. <https://doi.org/10.1016/S0168-583X(96)00676-3>.
4. Reimer P, Austin WEN, Bard E, Bayliss A, Blackwell PG, Bronk Ramsey C, et al. The IntCal20 Northern Hemisphere radiocarbon age calibration curve (0-55 cal kBP). Radiocarbon. 2020; 00: 1-33. DOI:10.1017/RDC.2020.41.
